# Alteration of platelet GPVI signaling in ST-elevation myocardial infarction patients demonstrated by a combination of proteomic, biochemical, and functional approaches

**DOI:** 10.1038/srep39603

**Published:** 2016-12-22

**Authors:** Paula Vélez, Raymundo Ocaranza-Sánchez, Diego López-Otero, Lilian Grigorian-Shamagian, Isaac Rosa, Esteban Guitián, José María García-Acuña, José Ramón González-Juanatey, Ángel García

**Affiliations:** 1 Center for Research in Molecular Medicine and Chronic Diseases (CIMUS), Universidade de Santiago de Compostela, Santiago de Compostela, Spain; 2 Instituto de Investigación Sanitaria de Santiago de Compostela (IDIS), Santiago de Compostela, Spain; 3 Cardiology Department and Coronary Care Unit, Hospital Clínico Universitario de Santiago, Santiago de Compostela, Spain; 4 Heart Institute, Cedars-Sinai Medical Center, Los Angeles, USA; 5 Mass Spectrometry and Proteomic Unit, Rede de Infraestructuras de Apoio á Investigación e ao Desenvolvemento Tecnolóxico (RIAIDT), Universidade de Santiago de Compostela, Santiago de Compostela, Spain

## Abstract

The platelet-specific collagen receptor glycoprotein VI (GPVI) is critical for the formation of arterial thrombosis *in vivo*. We analyzed GPVI-activated platelets from ST-elevation myocardial infarction (STEMI) patients and matched stable coronary artery disease (SCAD) controls in order to provide novel clues on the degree of involvement of GPVI signaling in the acute event. Firstly, platelets were isolated from systemic venous blood and activated with the GPVI specific agonist CRP (collagen-related peptide). STEMI and SCAD samples were compared by a phosphoproteomics approach. Validations were by immunoblotting in systemic and intracoronary blood from independent cohorts of patients. Twenty-six differentially regulated proteins were identified when comparing CRP-activated systemic platelets from STEMI and SCAD patients, 4 of which were selected for validation studies: PLCɣ2, G6f, SLP-76, and Dok-2. Immunoblot analyses showed these four proteins had higher tyrosine phosphorylation levels in response to CRP in platelets from STEMI patients, being these levels more pronounced at the culprit site of coronary artery occlusion. Moreover, platelet aggregation studies showed a higher response to GPVI agonists in STEMI patients compared to SCAD controls. In conclusion, we show an altered activation state of GPVI signaling in STEMI patients, confirming this receptor as a promising anti-thrombotic target for myocardial infarction.

ST-elevation myocardial infarction (STEMI) is a particular type of acute coronary syndrome (ACS) that involves intracoronary artery occlusion by a growing thrombus. Despite great progress done in the last decades in the treatment and follow up of patients with STEMI, the incidence of death is still high, being percutaneous coronary intervention (PCI) the most effective method to limit infarct size^1^.

It is well known that platelets have an imperative role in the development of acute coronary syndromes (ACS) since they attach to the vessel wall and initiate occlusion of the coronary vessel leading to myocardial ischemia, reason why they are considered as key players in myocardial infarction^2^. Specifically, the platelet collagen receptor glycoprotein VI (GPVI) triggers platelet activation and primary hemostasis. Several studies have demonstrated the crucial role of GPVI in arterial thrombosis *in vivo*^3^,^4^; indeed, it has been shown that platelet GPVI binds to collagenous structures in the core region of human atheromatous plaque^5^. The inhibition of this receptor results in protection against atherosclerosis in rabbit and mice models^6^ and also against thrombus formation on injured arteries^7^,^8^,^9^. Despite its crucial role in the process of arterial thrombosis, GPVI seems to be of minor importance for normal hemostasis, since its deficiency is not associated with any major bleeding risk in humans or mice^7^. All the aforementioned, together with the fact that GPVI is solely expressed in platelets and megakaryocytes, has made this receptor a promising antithrombotic target^10^.

In the present study, we aimed to check the activation state of GPVI signaling in STEMI patients by combining a tyrosine phosphoproteomics-based approach and immunoblotting-based validation assays. Platelets from STEMI patients and matched stable coronary artery disease (SCAD) controls were activated with the GPVI specific agonist *collagen-related peptide* (CRP). An initial proteomic approach led to the identification of a panel of GPVI signaling biomarkers hyperphosphorylated in STEMI that were validated by immunoblot on larger cohorts of patients. A complementary platelet aggregation-based validation approach was also undertaken. Moreover, GPVI signaling activation was also studied in platelets from intracoronary culprit-site arterial blood taken from STEMI patients during PCI compared to platelets from peripheral blood taken from the radial artery of the same patients. The latter was to test if platelets from blood in direct contact with the thrombus at the occluded coronary artery could reflect better the GPVI signaling activation levels compared to peripheral platelets; this would provide further clues on the relevance of this receptor in the pathological events associated with an acute myocardial infarction.

## Results

### Patients’ characteristics

For the venous blood studies, platelets from STEMI patients were compared to a control group of age- and gender-matched patients with SCAD. The matching was also based on treatments previous to blood extraction, paying special attention to anti-platelet treatment and statins. Venous and arterial blood samples were collected and platelets isolated as indicated in the Methods section. Besides the expected difference in having a previous history of CAD, there were no other significant differences between both groups, as shown in Table 1, 2 and 3 and Supplementary Table 1–3. For the arterial blood study, the characteristics of the STEMI patients were similar to those included in the venous study (see Table 4, and Supplementary Table 4).

### GPVI signaling proteomics in STEMI vs SCAD patients

**Figure 1:**
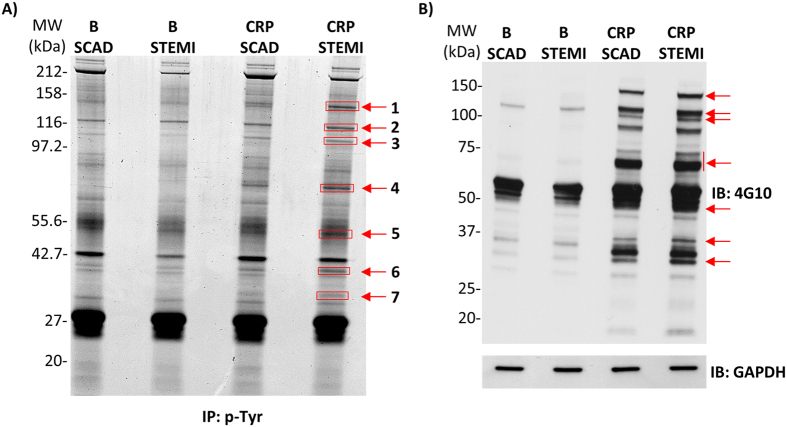
Tyrosine phosphoproteome analysis of GPVI signaling in platelets from STEMI and SCAD patients. (**A**) Total protein staining (SYPRO Ruby) of a 4–12% NuPAGE Bis-Tris gel showing proteins obtained after 4G10 mAb anti-phosphotyrosine immunoprecipitation of basal and CRP-stimulated platelets from STEMI patients (n = 5) and SCAD (n = 5) controls. 3 × 10^8^ platelets were used per immunoprecipitation. Equal amounts of protein corresponding to each of the five patients per group were pooled for each study point and loaded in different lanes of the gel. Indicated bands were excised from the gel and analyzed by MS. Proteins identified are reported in Table 5. An intense band in the 27 kDa region corresponds to IgG light chain. (**B**) Immunoblot analysis of p-Tyr (4G10 mAb), and GAPDH (loading control) protein expression levels using the same basal and CRP-activated platelet lysates than those used in (**A**), prior to immunoprecipitations. Equal amounts of protein corresponding to each of the five patients per group were pooled for each study point and loaded in different lanes in a 4–12% NuPAGE Bis-Tris gel for protein separation prior to western blotting. Regions with bands that could correlate with those highlighted in the stained gel are indicated. Basal, basal platelets; CRP, platelets stimulated with CRP (10 μg/mL, 90 sec), as indicated in the Methods section; IP, immunoprecipitation; IB, immunoblot.

Our initial goal was to analyze GPVI signaling activation by proteomics in venous blood platelets from STEMI patients in order to provide novel clues on the degree of involvement of this signaling cascade in the acute event. We decided to carry out a small scale proteomic analysis to choose a panel of GPVI activation biomarkers that could be used in larger cohorts of patients. To follow changes in GPVI activation levels only, platelets were activated with the GPVI specific agonist CRP under non aggregating conditions in the presence of inhibitors of secondary mediators. Since GPVI signaling goes primary through tyrosine phosphorylations, proteome analysis was based on immunoprecipitations using the agarose-conjugated 4G10 antiphosphotyrosine antibody, following the same strategy that was previously used to study the GPVI and CLEC-2 signaling cascades^11^,^12^. Proteome analysis involved samples from a group of 5 STEMI patients and 5 matched SCAD controls pooled independently (Table 1; Supplementary Table 1). P-Tyr proteome derived from basal- and CRP-stimulated platelets was resolved by 1D SDS-PAGE and the gel stained with a fluorescent dye (SYPRO Ruby). We focused on stained bands of higher intensity in the lane corresponding to CRP-stimulated platelets from STEMI patients, comparing to the equivalent SCAD control (Fig. 1A); those bands seemed to correlate with increased tyrosine phosphorylated bands in the immunoblot run in parallel (Fig. 1B), although such correlation is difficult to establish with total certainty due to intrinsic differences between both images (stained gel and immunoblot with and anti-p-Tyr antibody). In that way, 7 bands were manually excised for mass spectrometric analysis (Fig. 1A). As indicated above, an immunoblot analysis with whole platelet lysates was run in parallel using the 4G10 antiphosphotyrosine antibody and revealed an up-regulation of GPVI signaling in response to CRP in STEMI patients, which is reflected by an increase tendency in p-Tyr levels. GAPDH was used as a loading control (Fig. 1B). It is necessary to point out that Fig. 1B shows specific differences, so it is a valid reference to decide which bands to cut from the gel shown in Fig. 1A, although correlation between both gel images (stained gel and immunoblot) is not obvious. Indeed, Fig. 1B shows by immunoblot differences in tyrosine phosphorylation levels whereas Fig. 1A shows total protein gel staining, which means specific differences might be hidden by proteins recruited to the signaling complex that do not suffer changes in tyrosine phosphorylation in response to GPVI activation. Moreover, it is necessary to consider the presence of the “crapome”, which consists of an important number of unspecific proteins, pulled down during immunoprecipitations, and that can also hide specific differences^13^.

Mass spectrometric analysis of proteins present in the excised bands led to de identification of 26 unique proteins, including several well-known GPVI signaling proteins (Table 5; Supplementary Table 5; Supplementary Data). Those included the tyrosine kinase Syk, the adapters SLAP-130 and GADS, the enzyme PLCɣ2, the type I transmembrane protein G6f, and the adapters Dok-2, GADS, SLAP-130, and SLP-76.

### Platelet aggregation in response to GPVI stimulation is increased in STEMI patients compared to SCAD controls

**Figure 2:**
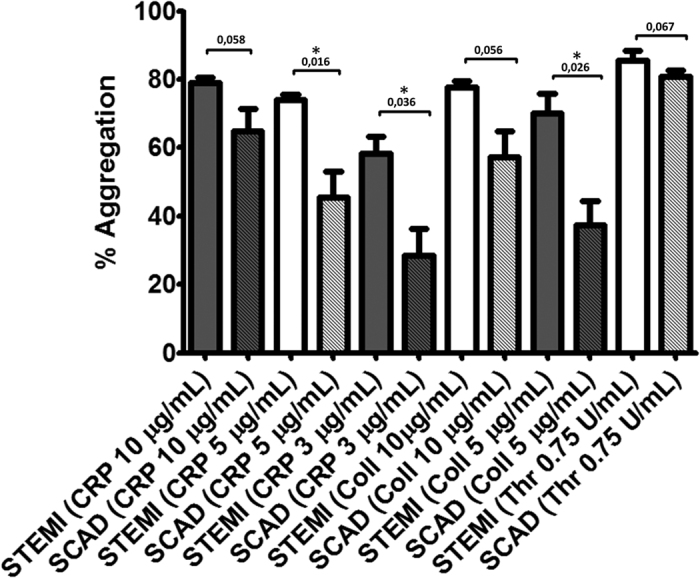
Platelet aggregation in response to GPVI activation is increased in STEMI patients compared to SCAD controls. Systemic venous-blood platelets were stimulated with Collagen (5 or 10 μg/mL), CRP (3, 5 or 10 μg/mL) or with thrombin (0.75 U/mL) to trigger aggregation. Results are presented as mean ± SE (n = 5). *p < 0.05 (Mann-Whitney test). Coll: collagen; Thr: thrombin.

In parallel to the proteomic study, we carried out a small scale analysis of platelet aggregation following activation with CRP and collagen at different doses. Thrombin was used as a positive control. For this analysis, platelets were obtained from an independent cohort of 5 STEMI patients and 5 matched SCAD controls (Table 2; Supplementary Table 2). In agreement with the biochemical data, platelet aggregation was higher in STEMI patients for CRP and collagen activations, with significant differences at doses of 5 μg/mL (Fig. 2). No significant differences in platelet aggregation between STEMI and SCAD were observed in the case of thrombin stimulations (Fig. 2). Following these results, we are currently planning an independent study to follow platelet aggregation responses to GPVI activation in larger cohorts of different subtypes of coronary artery disease patients.

### GPVI signaling is altered in systemic venous blood from STEMI patients

**Figure 3:**
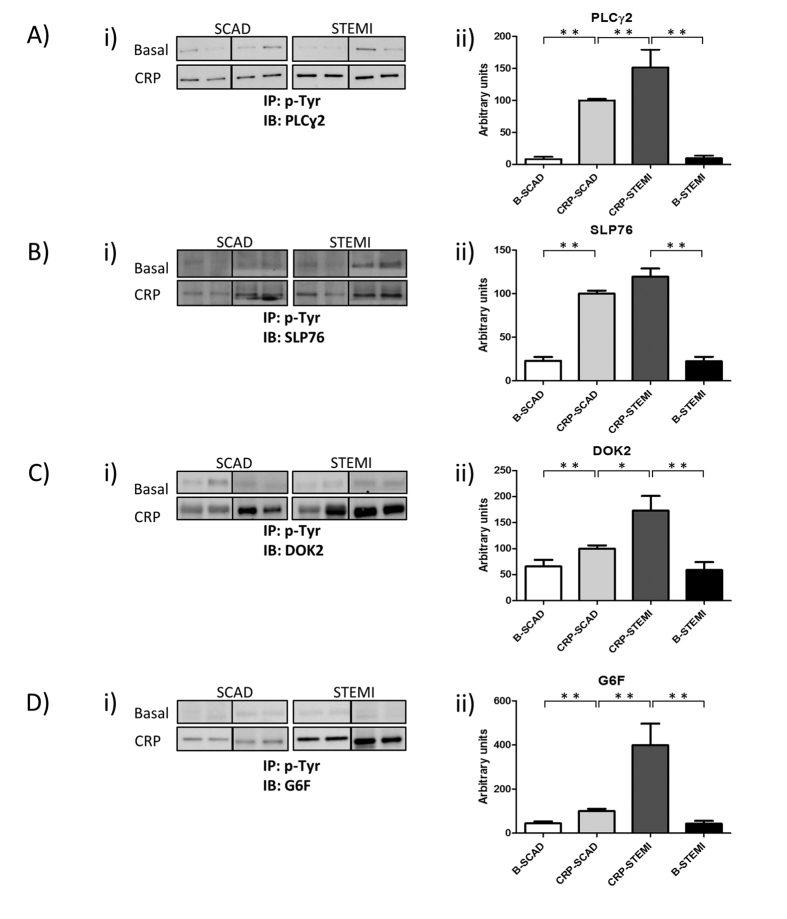
GPVI signaling activation is increased in systemic venous-blood platelets from STEMI patients compared to SCAD controls. Immunoblot analysis of PLCɣ2, SLP76, Dok-2, and G6f following immunoprecipitations with the anti-phosphotyrosine 4G10 mAb. STEMI and SCAD samples were compared. 2 × 10^8^ platelets were used per immunoprecipitation. (i) Representative images corresponding to individual patients (images for 4 STEMI patients and the corresponding SCAD controls are shown); (ii) Densitometry graphs representing average band intensities ± SE and statistics for all patients are shown (N_STEMI_:14; N_SCAD_: 11). All significant differences are indicated: *p < 0.05; **p < 0.01. Basal, basal platelets; CRP, platelets stimulated with CRP (10 μg/mL, 90 sec), as indicated in the Methods section; IP, immunoprecipitation; IB, immunoblot.

From the differentially regulated proteins identified by proteomics, we decided to choose a panel of four identified in bands showing more differences between groups and of clearly different molecular weight. The latter allowed us to maximize the limited amount of sample available because we could validate the proteins easier by immunoblotting after immunoprecipitations with the 4G10 antiphosphotyrosine antibody. The four proteins selected were (from higher to lower molecular weight): PLCɣ2, SLP-76, Dok-2, and G6f. We then validated those proteins in systemic venous blood platelets from 14 STEMI patients and 11 SCAD matched controls (Table 3; Supplementary Table 3). To follow GPVI activation levels, platelets were stimulated with the GPVI specific agonist CRP. We found a significant increase of p-Tyr for the four proteins when comparing basal and CRP-activated platelets (Fig. 3). However, when comparing CRP-activated platelets between groups, results showed increased p-Tyr levels in STEMI patients compared to SCAD for the four proteins, with significant increases in the case of PLCɣ2, Dok-2, and G6f (Fig. 3; Supplementary Tables 6–9). An increase in p-Tyr levels means increased GPVI signaling activation. The same number of platelets, and therefore protein amount, were used per immunoprecipitation.

### GPVI signaling activation is increased at the culprit site of coronary artery occlusion in STEMI patients

**Figure 4:**
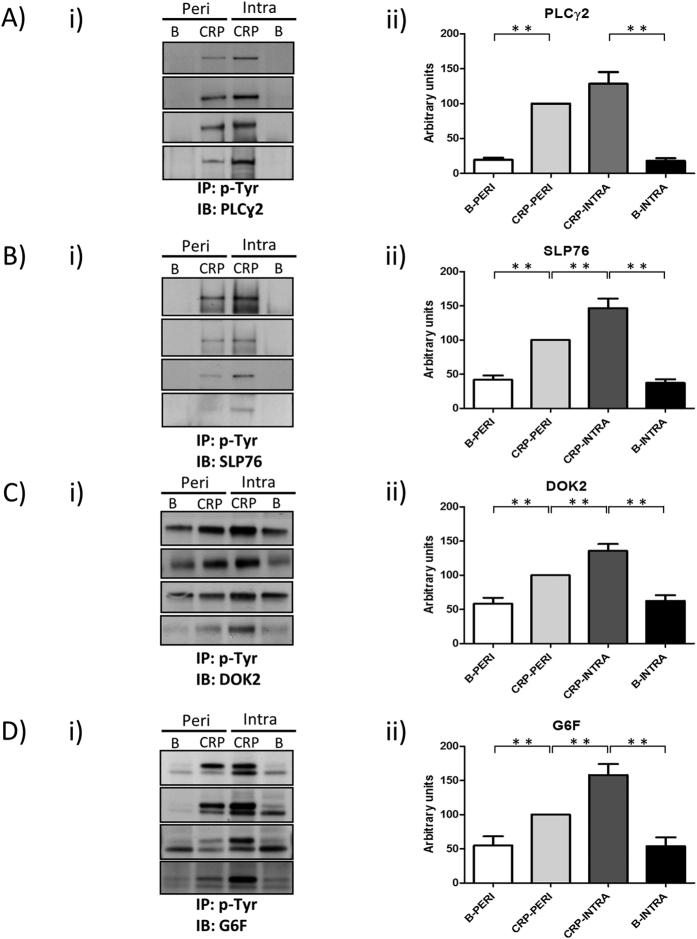
GPVI signaling activation is increased at the culprit site of coronary artery occlusion in STEMI patients. Platelets from arterial blood taken from the intracoronary culprit site - in contact with the thrombus at the occluded artery - and from the radial (peripheral) artery were compared. Immunoblot analysis of PLCɣ2, SLP76, Dok-2, and G6f following immunoprecipitations with the anti-phosphotyrosine 4G10 mAb are shown. 2 × 10^8^ platelets were used per immunoprecipitation. (i) Representative images corresponding to individual patients (four patients per panel); (ii) Densitometry graphs representing average band intensities ± SE and statistics for all patients are shown (N_STEMI_:16). A reference value of 100 is given to densitometry data of the CRP-PERI group. All significant differences are indicated: *p < 0.05; **p < 0.01. PERI, peripheral platelets from the radial artery; INTRA, intracoronary platelets from the culprit site. Basal, basal platelets; CRP, platelets stimulated with CRP (10 μg/mL, 90 sec), as indicated in the Methods section; IP, immunoprecipitation; IB, immunoblot.

To gain insight into the disease, we extended the initial venous blood study recruiting an additional cohort of 16 STEMI patients to check GPVI signaling activation in intracoronary arterial blood platelets by immunoblotting (Table 4; Supplementary Table 4). This is an innovative approach based on that platelets in direct contact with the thrombus could reflect better the increased levels of GPVI signaling activation observed in peripheral venous blood. To prove our hypothesis, blood platelets obtained from de intracoronary culprit site at the moment of reperfusion were compared with peripheral blood platelets taken from the radial artery through which the sheath was inserted. To follow GPVI activation levels, platelets were stimulated with CRP, as it happened in the venous study, followed by immunoprecipitations using the 4G10 antiphosphotyrosine antibody and immunoblotting with antibodies for the same set of proteins validated in the venous study. The same number of platelets, and therefore protein amount, were used per immunoprecipitation. Higher activation corresponds with an increase in p-Tyr levels. As expected, significant increases in p-Tyr levels were observed for all proteins when comparing basal and CRP-stimulated platelets (Fig. 4; Supplementary Tables 10–13). Interestingly, increased p-Tyr levels in response to CRP were observed in culprit site blood platelets, compared to CRP-activated peripheral arterial blood platelets, for the four selected signaling proteins. We found significant increases in p-Tyr levels in the case of the adapters SLP-76 and Dok-2 (Fig. 4B,C), and also for the transmembrane protein G6f (Fig. 4D). The increase of PLCɣ2 tyrosine phosphorylation was also evident in intracoronary samples after CRP stimulation, but nevertheless that tendency was not statistically significant (Fig. 4A). It is important to point out that in Fig. 4 each blot image represents a patient, where a reference value of 100 units in the densitometry is given to CRP-stimulated peripheral platelets.

## Discussion

The principal findings of the present study are: (i) proteomic and biochemical identification of a panel of tyrosine-phosphorylated signaling proteins reflecting altered GPVI activation levels in STEMI patients compared to SCAD matched controls; (ii) increased aggregation levels of CRP-, and collagen-activated platelets in STEMI patients, which is consistent with the data mentioned in the previous point; (iii) first GPVI signaling analysis of intracoronary and peripheral arterial platelets from STEMI patients, showing increased GPVI signaling activation levels in platelets at the culprit site of coronary artery occlusion.

Dual antiplatelet therapy is indicated in patients who have had ST-segment elevation myocardial infarction because it can safely and effectively reduce mortality and major adverse vascular events^14^. Nevertheless this treatment has the huge challenge of balancing thrombolysis and excessive blood loss. Since GPVI has a crucial role in arterial thrombosis *in vivo*^3^ and together with the fact that its deficiency is not associated with any major bleeding risk in humans or mice^7^, this receptor – for which there are no drugs in the market - is considered a hopeful target for the treatment of cardiovascular diseases providing a better risk/benefit ratio.

We have recently analyzed the proteome of platelets from ACS patients^15^,^16^, with special emphasis in STEMI, leading to the identification of a series of signaling proteins altered in the acute event. Several of these proteins are involved in integrin and GPVI signaling. Due to this fact and the relevance of GPVI mentioned above, we decided to focus on this target in the present study.

We chose a double approach to see GPVI activation levels in platelets from STEMI patients. Firstly, we studied systemic venous blood, which is the standard approach. A first group of patients was used to do a small-scale phosphoproteomic study that led to the selection of a panel of signaling proteins altered in STEMI that could be used to follow GPVI activation levels in STEMI patients and SCAD controls. We focused on the following four proteins:

1. The enzyme Phospholipase C gamma 2 (PLCγ2): key for the production of second messenger molecules diacylglycerol (DAG) and inositol 1,4,5-trisphosphate (IP3) in response to platelet collagen activation^10^;
2. The adapter SLP76: interacts with PLCγ2 to positively regulate GPVI signaling^10^;
3. The adapter docking protein 2 (Dok-2): involved in GPVI signaling^17^, where is primarily phosphorylated by Lyn kinase;
4. G6f, a type I transmembrane adapter protein belonging to the immunoglobulin superfamily, previously identified by us and others as being specifically phosphorylated on Tyr-281 in response to GPVI platelet activation, providing a docking site for the adapter Grb2^11^. Since this is the only tyrosine phosphorylation site on this protein, all differences observed in the present study related to G6f are due to differences in phosphorylation levels of Tyr-281.

The above proteins were studied in venous blood platelets by immunoblotting and we could demonstrate their tyrosine phosphorylation levels were increased in STEMI versus SCAD controls in response to the GPVI specific agonist CRP. Recent reports by Bigalke and collaborators demonstrated that GPVI surface expression is elevated in platelets from ACS patients^18^,^19^,^20^. More precisely, Bigalke and colleagues showed a clear increase of GPVI and P-selectin (CD62P) in the surface of ACS patients compared to SCAD, being this difference more evident in the case of STEMI patients^18^. Moreover, platelet GPVI correlated with P-selectin surface expression levels^18^. Although platelets have a limited synthesis capability, and there are several reports demonstrating the synthesis of various proteins in response to inflammatory and prothrombotic stimuli^21^,^22^, the increased GPVI surface expression in ACS is most probably due to a redistribution of GPVI internal pools to the surface, which would be triggered by the acute event or its imminence. This redistribution has been already shown in *in-vitro* GPVI activated platelets^23^. This increased GPVI surface expression is a good mechanistic explanation for the increased GPVI signaling activation in response to CRP we observe, demonstrating STEMI platelets can be activated through GPVI easier, compared to SCAD controls.

As an additional validation test, we carried out aggregation assays of platelets activated by GPVI agonists (CRP, and collagen) at different doses. Our results demonstrated an enhanced aggregation of platelets in response to both GPVI agonists in STEMI compared to SCAD controls, suggesting a higher activation of GPVI signaling in the former group, in line with our proteomic and immunoblotting data. It is interesting to mention that we recently showed that GPVI is the main receptor responsible for platelet aggregation by collagen^24^, so the similarity between CRP and collagen results makes sense. In these experiments, we used thrombin activations as a positive control of platelet viability; due to a sample limitation issue. That is the reason why we used only one dose, relatively high. In the near future we plan to carry out a specific aggregation study on STEMI patient subtypes checking several platelet receptors.

To further explore the involvement of GPVI in platelet activation associated with an STEMI, we analyzed tyrosine phosphorylation levels of the four biomarkers mentioned above in intracoronary arterial blood platelets, taken from the culprit site (i.e. in direct contact with the thrombus at the occluded artery) in comparison with platelets taken from a peripheral artery. There are precedents of intracoronary studies, using a similar approach, that allowed the detection of platelet activation biomarkers in the intracoronary blood. Indeed, we recently showed an up-regulation of signaling proteins in platelets from intracoronary blood at the culprit site - in comparison with peripheral platelets - in STEMI patients^25^, which highlights the relevance of studying platelets from occluded blood in direct contact with the thrombus as activation biomarkers are more prompted to be elevated in these platelets. As another example, Gresele and colleagues recently showed that platelets release matrix metealloproteinase-2 in the coronary circulation of patients with ACS as a consequence of platelet activation^26^. Thus, the study of the coronary circulation, and more precisely, of what happens at the culprit site, can give us further clues on platelet receptors that could be playing a relevant role in the acute event. When we tested our selected panel of GPVI signaling biomarkers in intracoronary blood platelets from STEMI patients, we found higher levels of tyrosine phosphorylation (reflecting activation) in response to CRP at the culprit site than in platelets from a peripheral artery (radial). We hypothesize that by studying platelets in direct contact with the thrombus we are somehow zooming into the culprit site to better observe biochemical events related to the acute episode. It is important to highlight, that in the arterial blood study each patient is its own control and platelets are just being compared from different sources (occluded intracoronary versus peripheral artery). Obviously, differences in this case are lower than when comparing venous blood platelets from STEMI and SCAD, where we are dealing with two different groups of patients. Our results with arterial blood platelets open a new line of investigation that will be pursued in larger cohorts of patients, and reinforce data from the venous study to strongly suggest a relevant role of GPVI signaling in platelet activation in STEMI.

### Study limitations

The present study has some limitations that should be considered. Sample limitation prevented us from doing more experiments in parallel, such as immunoprecipitations directly against the validated proteins, additional mass spec-based phosphomapping approaches, or carrying out complementary FACS analyses to check platelet activation markers. We are currently starting a new study with different subtypes of CAD patients to carry out further aggregations plus quantitative mass spec analyses to bypass limitations of western blot validations. Ideally, STEMI patients should have been with no medication but this was not possible for obvious reasons (they were given at least aspirin before arriving to hospital). To compensate for this factor, the control group consisted of SCAD patients with the same medication than the acute ones, trying to match in the best possible way all other clinical characteristics between groups.

In conclusion, by combining several approaches and studying intracoronary and systemic platelets, the present study demonstrates altered GPVI signaling activation levels in response to receptor stimulation in platelets from STEMI patients, confirming GPVI – and its signaling pathway - as promising anti-thrombotic targets for myocardial infarction.

## Materials and Methods

### Patients

The study was approved by the local Ethics Committee (Galician Investigation Ethics Committee) and developed according to the principles outlined in the Declaration of Helsinki. All methods were carried out in accordance with the approved guidelines, and informed consent was obtained from all subjects. Acute patients included in the study were admitted into a tertiary Spanish hospital presenting with STEMI, defined as angina pain of at least 20 minutes (min) duration with elevated cardiac enzymes and ST-segment elevation of at least 0.1 mV in two or more contiguous leads, or presumably new left bundle branch block. Exclusion criteria were inflammatory or neoplastic diseases, coagulation disorders, platelet-associated disorders, other significant heart disease except left ventricular hypertrophy secondary to hypertension, chronic drug therapy (except for drugs required to treat pre-existing clinical atherosclerosis or its risk factors), and having suffered from surgical procedures, major traumata, thromboembolic events or revascularization procedures in the previous three months. At the moment of diagnosis, 25 mL of venous blood or 50 mL of arterial blood (25 mL of intracoronary and 25 mL of peripheral) were collected from each patient in 3.2% sodium citrate Vacuette^®^ tubes for analysis. All samples were obtained within the first 12 hours following the initiation of the symptoms, after arrival at the emergency department. Most patients were administered aspirin and P2Y12 inhibitors, primarily clopidogrel, before the blood sample was taken (Tables 1, 2, 3 and 4).

For the venous study, besides the STEMI group, blood was also collected from a control group consisting of matched SCAD patients suffering from stable chronic ischemic cardiopathy defined by one of the following: 1) presence of stable angina with documented coronary artery disease (at least 70% stenosis in one of the coronary arteries; 2) history of coronary angioplasty; 3) history of coronary artery by-pass grafting; and, 4) history of acute coronary syndrome. Such a group was decided to be the most adequate control group due to the clinical characteristics and treatments of the STEMI patients. SCAD patients were required not to have a history of acute cardiovascular event within the year before the inclusion in the study.

Both STEMI and SCAD venous-blood was collected from cubital vein in plastic citrated tubes using 21-G needles. Intracoronary samples from STEMI patients were taken during normal hemodynamics procedure using a Pronto V3 catheter (Vascular Solutions), which occludes antegrade flow allowing collecting culprit site blood from the distal artery^25^,^27^,^28^. Peripheral arterial samples were obtained from the radial artery through which the sheath was inserted. Arterial blood samples were collected through the catheter into a syringe, and finally moved into plastic citrate tubes. Peripheral blood was collected first.

The initial venous blood proteomic screening included 5 STEMI patients and 5 SCAD matched controls (Table 1; Supplementary Table 1). Venous blood immunoblot validations included 14 STEMI patients and 11 SCAD matched controls (Table 3; Supplementary Table 3). An additional group of 5 STEMI patients and 5 SCAD matched controls was used for the platelet aggregation-based validation study (Table 2; Supplementary Table 2). In the arterial-derived blood study, an independent cohort of 16 STEMI patients was included (Table 4; Supplementary Table 4).

### Platelet agonists

The following agonists were used: Collagen-related peptide (CRP), with the sequence Gly-Cys-Hyp-(Gly-Pro-Hyp)_10_-Gly-Cys-Hyp-Gly-NH2, was provided by Dr. Richard W. Farndale, from the University of Cambridge (UK), and crosslinked with SPDP (3–2–pyridyldithio propionic acid N-hydroxysuccinimide ester) by Dr. Yotis A. Senis, at the University of Birmingham (UK). Collagen Reagent HORM^®^ Suspension (KRH) was purchased from Takeda Austria GmbH (Austria). Thrombin was purchased from Sigma (Sigma-Aldrich, St. Louis, MO).

### Platelet isolation, activation, and aggregation

Fresh blood samples were collected from STEMI patients and SCAD controls. Platelets were isolated by an established method in order to limit contamination from other blood cells and plasma proteins^29^. Finally washed platelets were resuspended in Tyrodes-HEPES (134 mmol/L NaCl, 0.34 mmol/L Na2HPO4, 2.9 mmol/L KCl, 12 mmol/L NaHCO3, 20 mmol/L HEPES, 5 mmol/L glucose, 1 mmol/L MgCl2, pH 7.3) and allowed to rest for 30 minutes at room temperature. For proteomic studies based on immunoprecipitations, concentrations of 6 × 10^8^ platelets/mL were used, whereas for immunodetection studies platelets were used at 4 × 10^8^ platelets/mL. Prior to stimulations, 1 mM EGTA, 10 μM indomethacin and 2 U/ml apyrase were added to washed platelets. For activations, 500-μL aliquots of platelets were warmed at 37 °C for 2 min without stirring and 30 sec with constant stirring at 1200 rpm in a Chrono-log aggregometer, before stimulation for 90 sec and constant stirring with CRP (10 μg/mL) or CRP diluent as control.

For aggregations, platelets were resuspended in Tyrodes-HEPES at a concentration of 3 × 10^8^ platelets/mL. No EGTA or inhibitors of secondary mediators (apyrase and indomethacin) were added. Following the resting step, 300-μL aliquots of platelets were warmed at 37 °C for 4 min without stirring and for 1 min with constant stirring at 1200 rpm in a Chrono-log aggregometer, before stimulation for 6 min with Collagen (5 or 10 μg/mL), CRP (3, 5 or 10 μg/mL) or with thrombin (0,75 U/mL).

### Immunoprecipitations and 1D-SDS-PAGE for proteomic analysis

Basal and stimulated platelets (6 × 10^8^/mL, 500 μL) were lysed with lysis buffer as described previously^16^. For the phosphotyrosine (P-Tyr) immunoprecipitations-based proteomic analysis, 10 μg of agarose-conjugated 4G10 monoclonal antiphosphotyrosine antibody (EMD Millipore Corporation, Billerica, MA, USA) were added to the lysates per immunoprecipitation and samples rotated overnight at 4 °C. Before the addition of the antibodies, samples were precleared with 25 μL of Protein A-Sepharose (50% w/v in TBS-T (20 mM Tris-HCl (pH 7.6), 137 mM NaCl, and 0.1% v/v Tween 20)) at 4 °C for 60 minutes with end-over-end mixing. After immunoprecipitations, proteins were eluted from the beads in 2X Laemmli sample buffer (4% w/v SDS, 10% v/v 2-mercaptoethanol, 20% v/v glycerol, 50 mM Tris, pH 6.8). Immunoprecipitations from five STEMI patients and five SCAD matched controls were pooled independently, and proteins resolved on 4–12% NuPAGE Bis-Tris gradient gels (Invitrogen, Carlsbad, CA, USA). After electrophoresis, the gel was fixed in 10% methanol/7% acetic acid for 1 hour, and stained overnight with SYPRO Ruby fluorescent dye. The gel was finally washed with 10% methanol/7% acetic acid for 30 min and scanned with a Bio-Rad Gel Doc XR system. Bands of interest were manually excised from the gel and in-gel digested with trypsin following the protocol defined by Shevchenko *et al*. with minor modifications^30^.

### Mass spectrometry analysis

Excised bands were identified by LC–MS/MS. Firstly, peptides were separated on an EASY-nLC (Proxeon, Bruker Daltonik GmbH) with a reverse phase nanocolumn (Easy column SC200 C18 3 μm 120 A 360 μm OD/75 μm ID, L = 10 cm) from Proxeon. Ionized peptides were analyzed in a Bruker Amazon ETD ion trap and database search was performed with the Mascot 2.3 search tool (Matrix Science, London, UK) screening SwissProt (SwissProt_2012_10.fasta). Further details on the LC-MS/MS protocol and MS protein identification data can be found in Supplementary Methods, Supplementary Table 5, and Supplementary Data.

### Immunoprecipitations and immunoblotting for immunodetection studies

Basal and CRP-stimulated platelets (4 × 10^8^/mL, 500 μL) were lysed as described before^17^ and immunoprecipitated. For immunoprecipitations related to immunoblotting experiments, 5 μg of agarose-conjugated 4G10 monoclonal antiphosphotyrosine antibody (from Millipore) were added to the lysates per immunoprecipitation. Immunoprecipitations and preclearings were done as indicated above. Immunoprecipitated proteins were eluted from the beads in 2X Laemmli sample buffer and separated in 10% SDS-PAGE gels or 4–12% NuPAGE Bis-Tris gradient gels (Invitrogen, Carlsbad, CA, USA). In some occasions, whole platelet lysates where also separated by SDS-PAGE. Following the electrophoresis, proteins were transferred onto polyvinylidene difluoride (PVDF) membranes (GE Healthcare). The membranes were blocked in 5% BSA in TBS-T (20 mM Tris-HCl (pH 7.6), 150 mM NaCl and 0.1% Tween 20) overnight at 4 °C and incubated for 90 min at room temperature with the following primary antibodies: mouse-anti PLCɣ2 (sc-5283), rabbit-anti SLP-76 (sc-9062), rabbit-anti Dok-2 (sc-13952), all of them from Santa Cruz Biotechnology, Inc. (Delaware, CA, USA); rabbit-anti 4G10 mouse antiphosphotirosine mAb (MAB1501) from Millipore; rabbit anti-GAPDH (G9545) from Sigma Aldrich; and G6f (1:1000), produced by CovalAB UK (Cambridge, UK), as previously indicated^11^. GAPDH immunoblots with the same lysates used for immunoprecipitation were used as a control to show there was no difference in the amount of protein immunoprecipitated. Following washes in TBS-T, membranes were exposed to horseradish peroxidase-labeled goat anti-mouse, or donkey anti-rabbit antibodies (dilutions 1:5000) (Pierce, Rockford, IL). Membranes were washed again and processed using an enhanced-chemiluminiscence system (ECL, Pierce, Rockford, USA) and quantified by densitometry. The densitometry analysis was represented as mean ± SE of all the independent experiments for each protein in each patient.

### Statistical analysis

Categorical variables from patients and controls are expressed as percentages and were compared using the Fisher exact test. Continuous variables are expressed as the median ± SD, and were compared by Mann-Whitney test.

In immunodetection studies by western blotting, densitometry of the bands were performed using ImageMeter version1.1 (Fashscript), and ImageJ (National Institute of Health, Bethesda, MD, USA) version 1.47, and statistical analysis was by Mann-Whitney test or Wilcoxon matched-pairs signed rank test comparing groups. All analyses were performed using IBM SPSS Statistics 20 software for Windows (IBM, Armonk, NY, USA) and graphs done with GraphPad Prism 5 (GraphPad Software, Inc. San Diego California, USA).

## Additional Information

**How to cite this article**: Vélez, P. *et al*. Alteration of platelet GPVI signaling in ST-elevation myocardial infarction patients demonstrated by a combination of proteomic, biochemical, and functional approaches. *Sci. Rep.* **6**, 39603; doi: 10.1038/srep39603 (2016).

**Publisher's note:** Springer Nature remains neutral with regard to jurisdictional claims in published maps and institutional affiliations.

## Supplementary Material

Supplementary Information

## Figures and Tables

**Table 1:** Clinical characteristics of STEMI and SCAD patients – Proteomics study.

Variable	STEMI (n = 5 Patients)	SCAD (n = 5 Patients)
Age, Y	64 ± SD 12	61 ± SD 16
Females, %	0	0
Hx arterial hypertension, %	40	40
Hx diabetes Miellitus, %	20	0
Hx dyslipidemia, %	60	60
Hx coronary artery disease, %*	20	100
Platelets/μL	269400 ± SD 114137.2	292000 ± SD 50642.9
Treatments		
Aspirin, %	100	100
Clopidogrel, %	100	100
Other antiplatelets, %	0	0
Anticoagulants, %	0	0

Further information can be found in Supplementary Table 1.

^*^p < 0.05.

**Table 2:** Clinical characteristics of STEMI and SCAD patients – Aggregation study.

Variable	STEMI (n = 5 Patients)	SCAD (n = 5 Patients)
Age, Y	73 ± SD 13	64 ± SD 15
Females, %	20	20
Hx arterial hypertension, %	20	20
Hx diabetes Miellitus, %	20	40
Hx dyslipidemia, %	20	60
Hx coronary artery disease, %*	0	100
Platelets/μL	239000 ± SD 41364.2	187000 ± SD 61110.6
Treatments		
Aspirin, %	80	100
Clopidogrel, %	60	60
Other antiplatelets, %	40	40
Anticoagulants, %	0	0

Further information can be found in Supplementary Table 2.

^*^p < 0.05.

**Table 3:** Clinical characteristics of STEMI and SCAD patients – Venous blood platelet immunoblotting study.

Variable	STEMI (n = 14 Patients)	SCAD (n = 11 Patients)
Age, Y	69 ± SD 14	64 ± SD 17
Females, %	21.4	9.1
Hx arterial hypertension, %	50	63.6
Hx diabetes Miellitus, %	35.7	18.2
Hx dyslipidemia, %	64.3	54.6
Hx coronary artery disease, %*	7.1	100
Platelets/μL	246538.5 ± SD 90981.6	244727.3 ± SD 57775.6
Treatments		
Aspirin, %	100	100
Clopidogrel, %	100	100
Other antiplatelets, %	0	0
Anticoagulants, %	0	0

Further information can be found in Supplementary Table 3.

^*^p < 0.05.

**Table 4:** Clinical characteristics of STEMI and SCAD patients – Arterial blood platelet immunoblotting study.

Variable	STEMI (n = 16 Patients)
Age, Y	67 ± SD 13
Females, %	37.5
Hx arterial hypertension, %	50
Hx diabetes Miellitus, %	31.3
Hx dyslipidemia, %	50
Hx coronary artery disease, %	6.3
Platelets/μL	243187.5 ± SD 79313.1
Treatments	
Aspirin, %	100
Clopidogrel, %	81.3
Other P2Y12 inhibitors, %	18.8
Anticoagulants, %	6.3

Further information can be found in Supplementary Table 4.

**Table 5:** Proteins identified in the proteomic study.

	Uniprot Code	Name
BAND 1	**PLCG2_HUMAN**	**Phospholipase C-gamma-2**
	ZO2_HUMAN	Tight junction protein
	MMRN1_HUMAN	Multimerin-1
BAND 2	FYB_HUMAN	FYN-binding protein (SLAP130)
	PK3CB_HUMAN	Phosphatidylinositol-4,5-bisphosphate 3-kinase catalytic subunit beta isoform
	ITA2B_HUMAN	Integrin alpha-IIb
BAND 3	ACTN1_HUMAN	Alpha-actinin-1
	FYB_HUMAN	FYN-binding protein (SLAP130)
	ACTN4_HUMAN	Alpha-actinin-4
BAND 4	SRC8_HUMAN	Src substrate cortactin
	**LCP2_HUMAN**	**Lymphocyte cytosolic protein 2 (SLP76)**
	KSYK_HUMAN	Tyrosine-protein kinase SYK
	HSP7C_HUMAN	Heat shock cognate 71 kDa protein
BAND 5	SKAP2_HUMAN	Src kinase-associated phosphoprotein 2
	**DOK2_HUMAN**	**Docking protein 2**
	TBA4A_HUMAN	Tubulin alpha-4A chain
	TBB1_HUMAN	Tubulin beta-1 chain
	TBB5_HUMAN	Tubulin beta-5 chain
	TBA3E_HUMAN	Tubulin alpha-3E chain
	ILK_HUMAN	Integrin-linked protein kinase
BAND 6	MK14_HUMAN	Mitogen-activated protein kinase 14
	GRAP2_HUMAN	GRB2-related adapter protein 2 (GADS)
	PPIP2_HUMAN	Proline-serine-threonine phosphatase-interacting protein 2
	ACTB_HUMAN	Actin, cytoplasmic 1
	PDLI1_HUMAN	PDZ and LIM domain protein 1
BAND 7	CAPZB_HUMAN	F-actin-capping protein subunit beta
	**LY66F_HUMAN**	**G6f**

Proteins identified in the 7 bands with increased intensity in CRP-activated STEMI platelets. Proteins selected for immunoblotting studies are highlighted in bold font.
