# Supplementary material for: Alteration of platelet GPVI signaling in ST-elevation myocardial infarction patients demonstrated by a combination of proteomic, biochemical, and functional approaches
Source: Sci Rep. 2016 Dec 22;6:39603. doi: 10.1038/srep39603 (PMC5177944; doi:10.1038/srep39603)
Supplement: Supplementary Information [file srep39603-s1.pdf]

Alteration of platelet GPVI signaling in ST-elevation myocardial infarction patients demonstrated by a combination of proteomic, biochemical, and functional approaches

Paula Vélez[1,2], Raymundo Ocaranza-Sánchez[2,3], Diego López-Otero[2,3], Lilian Grigorian-Shamagian[4], Isaac Rosa[1,2], Esteban Guitián[5], José María García-Acuña[2,3], José Ramón González-Juanatey[2,3], Ángel García*[1,2].

[1]Center for Research in Molecular Medicine and Chronic Diseases (CIMUS), Universidade de Santiago de Compostela, Santiago de Compostela, Spain; [2]Instituto de Investigación Sanitaria de Santiago de Compostela (IDIS), Santiago de Compostela, Spain; [3]Cardiology Department and Coronary Care Unit, Hospital Clínico Universitario de Santiago, Santiago de Compostela, Spain; [4]Heart Institute, Cedars-Sinai Medical Center, Los Angeles, USA; [5]Mass Spectrometry and Proteomic Unit, Rede de Infraestructuras de Apoio á Investigación e ao Desenvolvemento Tecnolóxico (RIAIDT), Universidade de Santiago de Compostela, Santiago de Compostela, Spain.

SUPPLEMENTARY INFORMATION

Supplementary Methods

Mass Spectrometric Analysis

Protein identifications were by LC-MS/MS. Digested peptide mixtures dissolved in 0.1% formic acid were separated in an EASY-nLC (Proxeon, Bruker Daltonik GmbH) with a reverse phase nanocolumn (Easy column SC20) from Proxeon (see Table 1 for more details).

Table 1.- LC parameters for separation of tryptic peptides

**LC settings**	
LC system	Easy-Nlc (PROXEON)
Trap column	Easy column SC001 C18-A1 5µm 120Å 360 µm OD/100µm ID, L=2cm
Analytical column	Easy column SC200 C18-A2 3µm 120Å 360 µm OD/75µm ID, L=10cm
Flow rate	300 nL/min
Eluents	A: 0.1% formic acid in water,<br>B:0.1% formic acid in acetonitrile
Gradient	5 to 35% B in 30 min, 35 to 100% B in 2 min, 100% B in 5 min, 100 to 5% B in 1 min, 5% B in 2 min

Ionized peptides were analyzed in an Amazon ETD ion trap mass spectrometer (Bruker Daltonics), equipped with a Nanosprayer ionization source that was used for data-dependent MS/MS experiments. Spectra were acquired in Enhanced Resolution mode. Further acquisition parameters are listed in Table 2.

Table 2.- MS and MS/MS settings used for acquisition with the Amazon ETD

**Acquisition parameters**	
Source	Nanosprayer®
MS settings:	
Scan mode	Enhanced resolution mode (8,100 m/z s^{-1})
Scan range	300-1,500 m/z
Spectra averages	5 (Rolling averaging: 1)
MS/MS settings:	
Scan mode	Enhanced resolution mode (8,100 m/z s^{-1})
Scan range	100-3,000 m/z
No. of precursor ions	3 (active exclusion after 1 spectrum, release after 0.2 min; reconsider precursor, if current intensity/previous intensity >1%
Isolation width	4 m/z
Spectral averages	2
Fragmentation amplitude (CID)	20% (SmartFrag 30-300%)
Fragmentation time (ETD)	100 ms

Automated analysis of mass data was achieved by Data Analysis 4.0 and BioTools 3.2 from Bruker Daltonik GmbH. Database search was performed with the Mascot v2.3 search tool (Matrix Science, London, UK) screening SwissProt (SwissProt_2012_10.fasta). Searches were restricted to human taxonomy allowing carbamidomethyl cysteine as a fixed modification and oxidized methionine and tyrosine phosphorylation as potential variable modification. Both the precursor mass tolerance and the MS/MS tolerance were set at 0.3 and 0.4 Da, respectively, allowing 1 missed tryptic cleavage site. All spectra and database results were manually inspected in detail using the above software, especially in the case of identifications based on one peptide hit. For the latter, positive identification by MS was only accepted when more than 50% y-ions (CID fragmentation) or z-ions (ETD fragmentation) were obtained for a peptide comprising at least eight amino acids long and no missed tryptic cleavage site. Positive hits corresponded to Mascot scores > 50 plus the fulfillment of the above criteria.

Supplementary Table 1. Clinical characteristics of STEMI and SCAD patients - proteomics study (expanded version). Data are presented as the median ± SD or percentage of patients. Abbreviations used: ACE: angiotensin converting enzyme; PTCA: percutaneous transluminal coronary angioplasty; * $p<0.05$.

**Variable**	**Acute Myocardial Infarction (N =5 patients)**	**Stable Coronary Artery Disease (N =5 patients)**
Age (years)	64 ± SD 12	61 ± SD 16
Females (%)	0	0
Hx Arterial Hypertension (%)	40	40
Hx Diabetes Mellitus (%)	20	0
Hx Smoking (%)	80	80
Hx Dyslipidemia (%)	60	60
Hx Coronary Artery Disease (%) *	20	100
Hx Cerebro-vascular Disease (%)	0	0
Hx Congestive Heart Failure (%)	0	0
Hx Peripheral Artery Disease (%)	0	0
Laboratory Measurements		
Hemoglobin (g/dl)	14.8 ± SD 1	13.6 ± SD 2.1
Leukocytes/µL	8888 ± SD 2366.3	9100 ± SD 3333.9
Platelets/µL	269400 ± SD 114137.2	292200 ± SD 50642.9
Mean Platelet Volume (fL)	8.5 ± SD 1.1	8.78 ± SD 0.7
Glucose (mg/dl)	140 ± SD 31.4	110.2 ± SD 34.8
Creatinin (mg/dl)	1 ± SD 0.3	1 ± SD 0.1
Cholesterol (mg/dl)	188 ± SD 59.4	161 ± SD 31.7
LDL-Cholesterol (mg/dl)	109.4 ± SD 57.1	78.6 ± SD 26.4
HDL-Cholesterol (mg/dl)	38.4 ± SD 15.3	47.8 ± SD 21.5
Triglycerides (mg/dl)	167 ± SD 110.7	166 ± SD 101.2
Chronic Treatments		
Aspirin (%)	100	100
Clopidogrel (%)	100	100
Other antiplatelets (%)	0	0
Anticoagulants (%)	0	0
ACE Inhibitors (%)	0	0
Angiotensin Receptor Blockers (%)	0	0
Statins (%)	80	80
Other		
Echocardiography (%)	100	100
Coronariography (%)	100	100
1-Vessel disease (%)	60	80
Left Descending Artery disease (%)	80	20
PTCA (%)	100	100

Supplementary Table 2. Clinical characteristics of STEMI and SCAD patients - venous blood platelet aggregation study (expanded version). Data are presented as the median ± SD or percentage of patients. Abbreviations used: ACE: angiotensin converting enzyme; PTCA: percutaneous transluminal coronary angioplasty; * p<0.05.

**Variable**	**Acute Myocardial Infarction (N =5 patients)**	**Stable Coronary Artery Disease (N =5 patients)**
Age (years)	73 ± SD 13	63.8 ± SD 15
Females (%)	20	20
Hx Arterial Hypertension (%)	20	20
Hx Diabetes Mellitus (%)	20	40
Hx Smoking (%)	60	40
Hx Dyslipidemia (%)	20	60
Hx Coronary Artery Disease (%) *	0	100
Hx Cerebro-vascular Disease (%)	0	0
Hx Congestive Heart Failure (%)	0	0
Hx Peripheral Artery Disease (%)	0	20
Laboratory Measurements		
Hemoglobin (g/dl)	13.5 ± SD 1.2	12.5 ± SD 2.2
Leukocytes/µL	9066 ± SD 4775.9	7196 ± SD 1346.3
Platelets/µL	239000 ± SD 41364.2	187000 ± SD 61110.6
Mean Platelet Volume (fL)	8.4 ± SD 2.0	8.8 ± SD 1.1
Glucose (mg/dl)	112.8 ± SD 26.7	101.2 ± SD 15.1
Creatinin (mg/dl)	1.0 ± SD 0.2	1.2 ± SD 0.7
Cholesterol (mg/dl)	163.6 ± SD 55.1	159.6 ± SD 87.0
LDL-Cholesterol (mg/dl)	98.6 ± SD 62.0	105.0 ± SD 70.1
HDL-Cholesterol (mg/dl)	37.6 ± SD 11.5	50.8 ± SD 15.4
Triglycerides (mg/dl)	138.0 ± SD 67.5	114.4 ± SD 24.4
Chronic Treatments		
Aspirin (%)	80	100
Clopidogrel (%)	60	60
Other antiplatelets (%)	40	40
Anticoagulants (%)	0	0
ACE Inhibitors (%)	80	100
Angiotensin Receptor Blockers (%)	100	100
Statins (%)	20	20
Other		
Echocardiography (%)	100	80
Coronariography (%)	100	100
1-Vessel disease (%)	100	100
Left Descending Artery disease (%)	100	80
PTCA (%)	100	60

Supplementary Table 3. Clinical characteristics of STEMI and SCAD patients - venous blood immunoblotting study (expanded version). Data are presented as the median ± SD or percentage of patients. Abbreviations used: ACE: angiotensin converting enzyme; PTCA: percutaneous transluminal coronary angioplasty; * p<0.05.

**Variable**	**Acute Myocardial Infarction (N =14 patients)**	**Stable Coronary Artery Disease (N =11 patients)**
Age (years)	69 ± SD 14	64 ± SD 17
Females (%)	21.4	9.09
Hx Arterial Hypertension (%)	50	63.6
Hx Diabetes Mellitus (%)	35.6	18.2
Hx Smoking (%)	78.6	72.7
Hx Dyslipidemia (%)	64.3	54.6
Hx Coronary Artery Disease (%) *	7.1	100
Hx Cerebro-vascular Disease (%)	0	0
Hx Congestive Heart Failure (%)	0	0
Hx Peripheral Artery Disease (%)	0	0
Laboratory Measurements		
Hemoglobin (g/dl)	14.5 ± SD 14.2	14.5 ± SD 1.8
Leukocytes/µL	8767.7 ± SD 2209.6	8480.9 ± SD 2499.4
Platelets/µL	246538.5 ± SD 90981.6	244727.3 ± SD 57775.6
Mean Platelet Volume (fL)	8.3 ± SD 1	8.5 ± SD 1
Glucose (mg/dl)	147.3 ± SD 37.1	119.7 ± SD 39.9
Creatinin (mg/dl)	0.95 ± SD 0.2	1 ± SD 0.3
Cholesterol (mg/dl)	159.4 ± SD 47.4	169.7 ± SD 30.9
LDL-Cholesterol (mg/dl)	84.6 ± SD 42.5	86.8 ± SD 23.5
HDL-Cholesterol (mg/dl)	37.1 ± SD 13.7	42.1 ± SD 15.5
Triglycerides (mg/dl)	185.2 ± SD 178	203.5 ± SD 121.4
Chronic Treatments		
Aspirin (%)	100	100
Clopidogrel (%)	100	100
Other antiplatelets (%)	0	0
Anticoagulants (%)	0	0
ACE Inhibitors (%)	7.1	0
Angiotensin Receptor Blockers (%)	0	9.1
Statins (%)	71.4	72.7
Other		
Echocardiography (%)	100	100
Coronariography (%)	100	100
1-Vessel disease (%)	64.3	90.9
Left Descending Artery disease (%)	42.9	36.4
PTCA (%)	100	100

Supplementary Table 4. Clinical characteristics of STEMI patients - arterial blood study (expanded version). Data are presented as the median ± SD or percentage of patients. Abbreviations used: ACE: angiotensin converting enzyme; PTCA: percutaneous transluminal coronary angioplasty; * $p<0.05$.

**Variable**	**Acute Myocardial Infarction (N =16 patients)**
Age (years)	67 ± SD 13
Females (%)	37.5
Hx Arterial Hypertension (%)	50
Hx Diabetes Mellitus (%)	31.3
Hx Smoking (%)	37.5
Hx Dyslipidemia (%)	50
Hx Coronary Artery Disease (%)	6.3
Hx Cerebro-vascular Disease (%)	0
Hx Congestive Heart Failure (%)	0
Hx Peripheral Artery Disease (%)	0
Laboratory Measurements	
Hemoglobin (g/dl)	12.7 ± SD 2.9
Leukocytes/µL	11296.3 ± SD 5590.3
Platelets/µL	243187.5 ± SD 79313.1
Mean Platelet Volume (fL)	8.5 ± SD 1.3
Glucose (mg/dl)	126.3 ± SD 48.9
Creatinin (mg/dl)	1 ± SD 0.7
Cholesterol (mg/dl)	162.9 ± SD 39.8
LDL-Cholesterol (mg/dl)	93.5 ± SD 33.7
HDL-Cholesterol (mg/dl)	38.6 ± SD 12.8
Triglycerides (mg/dl)	159.7 ± SD 57.3
Chronic Treatments	
Aspirin (%)	100
Clopidogrel (%)	81.3
Other antiplatelets (%)	18.8
Anticoagulants (%)	6.3
ACE Inhibitors (%)	18.8
Angiotensin Receptor Blockers (%)	12.5
Statins (%)	87.5
Other	
Echocardiography (%)	100
Coronariography (%)	100
1-Vessel disease (%)	68.8
Left Descending Artery disease (%)	56.3
PTCA (%)	100

Supplementary Table 5. Detailed information on MS protein identification

	Uniprot Code	Full Name	Score	MW theoretical	pI theoretical	Peptides (modifications)	Sequence Coverage
**BAND 1**	PLCG2_HUMAN	1-phosphatidylinositol-4,5-bisphosphate phosphodiesterase gamma-2	435.3	147.8	6.2	KQIYSVDQTR<br>KLMFEQQK (Oxidation: 3)<br>SILDEFKK<br>GEAEDMLMR (Oxidation: 6, 8)<br>EGSDSYAITFR<br>DINSLYDVSR (Phospho: 6)<br>GALIHNVSK<br>TKDNLENPDFR<br>TGYVLQPESMR (Oxidation: 10)	6.9
	ZO2_HUMAN	Tight junction protein	224.6	133.9	7.1	AEQMASVQNAQR (Oxidation: 4)<br>SNPSAVAGNETPGASTK<br>MQELQEAQNAR (Oxidation: 1)	3.4
	MMRN1_HUMAN	Multimerin-1	173.8	138.0	9.0	MTDQVNYQAMK (Oxidation: 1, 10)<br>QTMTYEQPK (Oxidation: 3)<br>NEVQGRDDALER<br>LQDIESK	3.2
**BAND 2**	FYB_HUMAN (SLAP130)	FYN-binding protein	772.3	85.3	6.1	VTGPNSSSGIQAR<br>EPKPPFLKPTGAGQR<br>SGPTPPTSENEQK<br>GSPAPLGVR<br>GGPGLSKNGEEK<br>INQEELASGTPPAR<br>GGKNELSFK<br>QGEQIEIIR<br>GSYGYIK<br>TTAVEIDYDSLK<br>YDGEIR<br>VLYSTK<br>VTTSITSK	17.0
	PK3CB_HUMAN	Phosphatidylinositol-4,5-bisphosphate 3-kinase catalytic subunit beta isoform	89.7	122.7	6.7	AAEIASSDSANVSSR	1.4
	ITA2B_HUMAN	Integrin alpha-IIb	57.2	113.3	5.1	NVGSQTLQTFK	1.1
**BAND 3**	ACTN1_HUMAN	Alpha-actinin-1	630.6	103.0	5.1	ALDFIASK<br>HRPELIDYGK<br>MLDAEDIVGTARPDEK (Oxidation: 1)	13.1

						LSNRPAFMPSEGR<br>ATLPDADKER<br>LAILGIHNEVSK<br>RDQALTEEHAR<br>DQALTEEHAR<br>HTNYTMEHIR (Oxidation: 6)<br>GISQEQMNEFR (Oxidation: 7)<br>ETADTDTADQVMASFK (Oxidation: 12)	
	FYB_HUMAN (SLAP130)	FYN-binding protein	145.4	85.3	6.1	VTGPNSSSGIQAR<br>SGPTPPTSENEQK<br>QGEQIEIIR	4.5
	ACTN4_HUMAN	Alpha-actinin-4	141.3	104.8	5.2	ALDFIASK<br>VQQLVPK<br>HTNYTMEHIR (Oxidation: 6)	2.7
**BAND 4**	SRC8_HUMAN	Src substrate cortactin	1081.4	61.5	5.1	TVQGSGHQEHINIHK<br>LRENVFQEHQTLK<br>ENVFQEHQTLK<br>FGVEQDRMDK (Oxidation: 8)<br>FGVQMDR (Oxidation: 5)<br>YGVQADR<br>YGVQADRVDK<br>SAVGFDYQGK<br>VDKSAVGFEYQGK<br>SAVGFEYQGK<br>FGVQTDR<br>LQLHESQK<br>FGVQSER<br>MDKNASTFEDVTQVSSAYQK (Oxidation: 1)<br>NASTFEDVTQVSSAYQK<br>TVPVEAVTSK<br>ANFENLAK<br>LPSSPVYEDAASFK (Phospho: 7)<br>GPVSGTEPEPVYSMEAADYR (Oxidation: 14; Phospho: 12)<br>GPVSGTEPEPVYSMEAADYR (Oxidation: 14)	31.3
	LCP2_HUMAN (SLP76)	Lymphocyte cytosolic protein 2	893.8	60.2	5.9	KYHIDGAR<br>FLNLTENDIQK<br>LSQEINKNEER<br>TPQQPPVPPQRPMAALPPPPAGR (Oxidation: 13)<br>NHSPLPPPQTNHEEPSR<br>LPAPSIDR<br>SLAPFDREPFTLGK<br>KPPFSDKPSIPAGR	33.0

						IQKPPLPPTTERHER<br>KINQDGTFLVR<br>INQDGTFLVR<br>TTTNPYVLMVLYK (Oxidation:9)<br>DKVYNIQIR<br>VYNIQIR<br>ESQVYLLGTGLR<br>KMPLLLIDGK (Oxidation: 2)<br>MPLLLIDGK (Oxidation: 1)	
	KSYK_HUMAN (SYK72)	Tyrosine-protein kinase SYK	353.2	72.0	9.1	ELNGTYAIAGGR<br>LIATTAHEK<br>ISREESEQIVLIGSK<br>EESEQIVLIGSK<br>ISDFGLSK	8.8
	HSP7C_HUMAN	Heat shock cognate 71 kDa protein	189.8	70.9	5.2	TTPSYVAFTDTER<br>VQVEYKGETK<br>NSLESYAFNMK (Oxidation: 10)	5.3
**BAND 5**	SKAP2_HUMAN	Src kinase-associated phosphoprotein 2	258.2	41.2	4.4	SIYLQEFQDK<br>TVFYYYGSDKDK<br>IYQFTAASPK<br>RGDVIYILSK<br>GDVIYILSK	11.7
	DOK2_HUMAN	Docking protein 2	161.8	45.4	5.7	VAEAGGEASSPR<br>DTSAFFLETK	5.3
	TBA4A_HUMAN	Tubulin alpha-4A chain	121.8	49.9	4.8	EIIDPVLDR<br>DVNAAIAAIK	4.2
	TBB1_HUMAN	Tubulin beta-1 chain	103.7	50.3	4.9	GASALQLER<br>LAVNMVPFPR (Oxidation: 5)	4.2
	TBB5_HUMAN	Tubulin beta chain	93.2	49.6	4.6	LAVNMVPFPR (Oxidation: 5)<br>MSMKEVDEQMLNVQNK (Oxidation: 1,3,10)	5.9
	TBA3E_HUMAN	Tubulin alpha-3E chain	81.5	49.8	4.9	DVNAAIATIK	2.2
	ILK_HUMAN	Integrin-linked protein kinase	63.4	51.4	9.3	SVMIDEDMTAR (Oxidation: 3,8)	2.4
**BAND 6**	MK14_HUMAN	Mitogen-activated protein kinase 14	550.7	41.3	5.4	YIHSADIIHR<br>ILDFGLAR<br>HTDDEMTGYVATR (Oxidation: 6; Phospho: 7)<br>HTDDEMTGYVATR (Oxidation: 6)<br>HTDDEMTGYVATR (Oxidation: 6; Phospho: 9)<br>LVGTPGAELLK<br>LVGTPGAELLKK<br>NYIQSLTQMPK (Oxidation: 9)<br>MLVLDSDKR (Oxidation: 1)	19.7

						DLLIDEWK	
	GRAP2_HUMAN (GADS)	GRB2-related adapter protein 2	337.6	37.9	6.5	HQAENLLMGK (Oxidation 8)<br>EVGFFIIR<br>ASQSSPGDFSISVR<br>HEDDVQHFK<br>RHTDPVQLQAAGR<br>HTDPVQLQAAGR	16.4
	PPIP2_HUMAN	Proline-serine-threonine phosphatase-interacting protein 2	262.5	38.8	9.5	YGKDLLNLSR<br>DKDEAEQAVSR<br>SANLVNPK<br>DIEYFVNQR<br>ATGPNLAR	13.8
	ACTB_HUMAN	Actin, cytoplasmic 1	240.4	41.7	5.2	HQGVMVGMGQK (Oxidation: 5,8)<br>DSYVGDEAQSK<br>EITALAPSTMK (Oxidation: 10)<br>QEYDESGPSIVHR	12.3
	PDLI1_HUMAN	PDZ and LIM domain protein 1	108.0	36.0	6.6	SAMPFTASPASSTTAR (Oxidation: 3)<br>VAASIGNAQK	7.9
**BAND 7**	CAPZB_HUMAN	F-actin-capping protein subunit beta	122.2	31.3	5.2	SGSGTMNLGGSLTR (Oxidation: 6)<br>LVEDMENKIR (Oxidation: 5)	8.7
	LY66F_HUMAN (G6F)	Lymphocyte antigen 6 complex locus protein G6f	40.0 (*)	32.4	10.1	VYDVLVLK	2.7

*Score<50 but validated.

Supplementary Table 6. PLCγ2 densitometric values from immunoblot analyses of systemic venous-blood platelets from STEMI patients compared to SCAD controls (see Figure 3). Values are in arbitrary units. Samples were distributed between several gels where STEMI patients were run with their SCAD controls, giving a reference value of 100 to the average SCAD-CRP value per gel.

**PLCγ2**				
**SCAD BASAL**	**SCAD CRP**	**STEMI CRP**	**STEMI BASAL**	
4.02	98.44	124.79	4.60	
5.04	106.21	108.18	7.50	
0.74	95.35	119.43	10.03	
2.37	100.00	123.47	0.77	
17.47	100.00	121.19	0.47	
39.87	100.00	168.34	30.98	
4.14	82.98	480.30	54.02	
10.48	117.02	134.13	4.88	
3.09	100.00	132.84	5.07	
1.36	100.00	150.13	3.31	
3.46	100.00	131.12	3.48	
		223.90	8.11	
		73.21	0.27	
		30.58	4.01	
**8.367**	**100.0**	**151.5**	**9.821**	**MEAN**
**3.468**	**2.416**	**27.81**	**3.965**	**SEM**
**11.50**	**8.014**	**104.1**	**14.84**	**SD**

Supplementary Table 7. SLP76 densitometric values from immunoblot analyses of systemic venous-blood platelets from STEMI patients compared to SCAD controls (see Figure 3). Values are in arbitrary units. Samples were distributed between several gels where STEMI patients were run with their SCAD controls, giving a reference value of 100 to the average SCAD-CRP value per gel. In some occasions the same SCAD control was valid for two STEMI patients and run with each one in different gels.

SLP76				
**SCAD BASAL**	**SCAD CRP**	**STEMI CRP**	**STEMI BASAL**	
14.45	111.54	102.35	9.79	
5.44	97.83	99.20	8.50	
28.82	90.63	90.15	59.12	
54.69	100.00	114.30	10.85	
46.29	100.00	118.12	35.43	
22.81	100.00	100.70	66.24	
26.10	124.75	134.99	17.14	
27.06	75.25	128.28	4.20	
12.89	100.00	169.53	19.67	
3.04	100.00	194.77	32.81	
12.16	100.00	162.41	18.25	
21.17	100.00	81.64	8.83	
		84.41	6.32	
		91.22	16.56	
**22.91**	**100.0**	**119.4**	**22.41**	**MEAN**
**4.446**	**3.315**	**9.300**	**5.186**	**SEM**
**15.40**	**11.48**	**34.80**	**19.40**	**SD**

Supplementary Table 8. DOK2 densitometric values from immunoblot analyses of systemic venous-blood platelets from STEMI patients compared to SCAD controls (see Figure 3). Values are in arbitrary units. Samples were distributed between several gels where STEMI patients were run with their SCAD controls, giving a reference value of 100 to the average SCAD-CRP value per gel. In some occasions the same SCAD control was valid for two STEMI patients and run with each one in different gels.

DOK2				
**SCAD BASAL**	**SCAD CRP**	**STEMI CRP**	**STEMI BASAL**	
64.10	80.79	184.97	47.97	
56.16	59.68	111.41	35.02	
58.22	159.53	182.66	63.64	
88.88	100.00	126.49	32.90	
12.82	100.00	48.22	12.79	
4.14	104.95	420.02	125.43	
110.70	95.05	305.41	212.20	
41.09	120.66	156.63	16.42	
69.75	79.34	146.15	21.98	
42.20	100.00	300.22	23.50	
187.80	100.00	167.40	37.66	
51.67	100.00	53.20	30.18	
66.99	100.00	59.43	30.75	
		162.49	133.54	
**65.73**	**100.0**	**173.2**	**58.86**	**MEAN**
**12.80**	**6.426**	**28.21**	**15.46**	**SEM**
**46.17**	**23.17**	**105.6**	**57.85**	**SD**

Supplementary Table 9. G6F densitometric values from immunoblot analyses of systemic venous-blood platelets from STEMI patients compared to SCAD controls (see Figure 3). Values are in arbitrary units. Samples were distributed between several gels where STEMI patients were run with their SCAD controls, giving a reference value of 100 to the average SCAD-CRP value per gel. In some occasions the same SCAD control was valid for two STEMI patients and run with each one in different gels.

G6F				
**SCAD BASAL**	**SCAD CRP**	**STEMI CRP**	**STEMI BASAL**	
76.66	83.39	325.75	48.50	
66.64	123.59	517.52	49.38	
50.88	93.01	468.14	68.73	
66.14	100.00	1425.65	24.21	
27.15	100.00	302.16	25.45	
95.92	153.00	824.89	156.44	
92.70	47.00	582.51	131.12	
18.51	33.72	287.43	15.74	
23.08	166.28	121.69	20.42	
12.93	100.00	222.80	17.36	
15.37	84.72	205.32	13.95	
19.27	115.28	53.46	13.31	
13.20	100.00	101.54	13.80	
		148.70	7.67	
**44.50**	**100.0**	**399.1**	**43.29**	**MEAN**
**8.733**	**10.05**	**97.53**	**12.35**	**SEM**
**31.49**	**36.23**	**364.9**	**46.21**	**SD**

Supplementary Table 10. PLCγ2 densitometric values from immunoblot analyses of arterial-blood platelets from STEMI patients (see Figure 4). Platelets from blood taken from the intracoronary culprit site and from radial (peripheral) arterial blood were compared. Values are in arbitrary units, giving for each patient a 100 value to peripheral CRP samples.

PLCγ2				
PERIPHERAL BASAL	PERIPHERAL CRP	INTRACORONARY CRP	INTRACORONARY BASAL	
53.06	100.00	356.62	24.49	
28.32	100.00	122.14	12.46	
21.36	100.00	174.96	66.17	
13.05	100.00	113.79	13.78	
20.07	100.00	90.24	16.43	
13.05	100.00	159.30	10.94	
26.23	100.00	114.68	35.26	
6.01	100.00	80.13	23.83	
12.64	100.00	92.24	17.28	
15.95	100.00	99.15	8.12	
40.71	100.00	108.29	11.10	
11.46	100.00	119.30	5.90	
4.50	100.00	158.36	3.05	
3.50	100.00	91.20	9.79	
19.26	100.00	89.28	8.16	
20.27	100.00	85.62	21.28	
**19.34**	**100.00**	**128.46**	**18.00**	**MEAN**
**3.275**	**0.0**	**16.80**	**3.811**	**SEM**
**13.10**	**0.0**	**67.19**	**15.19**	**SD**

Supplementary Table 11. SLP76 densitometric values from immunoblot analyses of arterial-blood platelets from STEMI patients (see Figure 4). Platelets from blood taken from the intracoronary culprit site and from radial (peripheral) arterial blood were compared. Values are in arbitrary units, giving for each patient a 100 value to peripheral CRP samples.

**SLP76**				
**PERIPHERAL BASAL**	**PERIPHERAL CRP**	**INTRACORONARY CRP**	**INTRACORONARY BASAL**	
114.04	100.00	275.11	69.98	
56.00	100.00	134.03	69.77	
30.22	100.00	197.63	21.34	
23.55	100.00	122.40	20.78	
19.79	100.00	86.54	44.21	
32.79	100.00	102.68	32.05	
36.09	100.00	119.51	13.01	
22.76	100.00	160.66	10.63	
28.84	100.00	218.01	43.51	
59.87	100.00	141.70	38.88	
38.45	100.00	107.32	27.62	
64.28	100.00	194.25	26.84	
10.49	100.00	209.73	17.48	
19.74	100.00	96.14	24.71	
56.98	100.00	97.44	83.42	
52.96	100.00	81.04	50.66	
**41.68**	**100.00**	**146.51**	**37.18**	**MEAN**
**6.351**	**0.0**	**14.22**	**5.450**	**SEM**
**25.37**	**0.0**	**56.88**	**21.80**	**SD**

Supplementary Table 12. DOK2 densitometric values from immunoblot analyses of arterial-blood platelets from STEMI patients (see Figure 4). Platelets from blood taken from the intracoronary culprit site and from radial (peripheral) arterial blood were compared. Values are in arbitrary units, giving for each patient a 100 value to peripheral CRP samples.

DOK2				
**PERIPHERAL BASAL**	**PERIPHERAL CRP**	**INTRACORONARY CRP**	**INTRACORONARY BASAL**	
142.00	100.00	203.96	116.50	
103.33	100.00	156.47	107.23	
43.68	100.00	191.67	83.66	
80.59	100.00	126.01	100.68	
44.06	100.00	204.38	51.48	
38.35	100.00	101.85	39.06	
39.54	100.00	135.13	28.50	
22.70	100.00	163.89	111.54	
29.54	100.00	136.62	38.32	
72.60	100.00	115.61	54.00	
40.25	100.00	108.61	63.53	
99.99	100.00	154.51	69.90	
73.36	100.00	110.48	42.35	
7.80	100.00	74.12	9.03	
37.05	100.00	90.29	31.92	
55.88	100.00	95.30	54.81	
**58.17**	**100.00**	**135.56**	**62.66**	**MEAN**
**8.731**	**0.0**	**10.12**	**8.172**	**SEM**
**34.85**	**0.0**	**40.52**	**32.68**	**SD**

Supplementary Table 13. G6F densitometric values from immunoblot analyses of arterial-blood platelets from STEMI patients (see Figure 4). Platelets from blood taken from the intracoronary culprit site and from radial (peripheral) arterial blood were compared. Values are in arbitrary units, giving for each patient a 100 value to peripheral CRP samples.

**G6F**				
**PERIPHERAL BASAL**	**PERIPHERAL CRP**	**INTRACORONARY CRP**	**INTRACORONARY BASAL**	
160.07	100.00	288.32	97.05	
159.35	100.00	197.47	135.80	
63.14	100.00	233.52	74.72	
137.27	100.00	208.65	186.63	
58.16	100.00	146.31	78.67	
39.80	100.00	107.42	44.89	
16.05	100.00	224.19	7.18	
8.66	100.00	164.64	73.65	
38.44	100.00	139.49	56.90	
20.64	100.00	119.41	3.87	
9.58	100.00	102.66	8.12	
95.95	100.00	224.78	8.93	
4.63	100.00	130.86	0.74	
7.93	100.00	89.24	11.97	
27.36	100.00	74.86	25.38	
31.77	100.00	76.58	44.58	
**54.92**	**100.00**	**158.03**	**53.69**	**MEAN**
**13.53**	**0.0**	**16.11**	**13.23**	**SEM**
**53.96**	**0.0**	**64.44**	**52.92**	**SD**

Supplementary Data. Mascot search results with annotated MS/MS spectra corresponding to protein identifications based on one peptide hit.

Mascot Search Results

Peptide View

MS/MS Fragmentation of **NVGSQTLQTFK**
Found in **ITA2B_HUMAN**, Integrin alpha-IIb OS=Homo sapiens GN=ITGA2B PE=1 SV=3

Match to Query 404: 1221.566624 from(611.790588,2+) intensity(487252.0000)
Title: Cmpd 192, +MSn(611.7906), 17.5 min (572657)
Local Instrument: ESI-TRAP
Data file 1247.mgf

Click mouse within plot area to zoom in by factor of two about that point
Or, Plot from 100 to 1100 Da Full range
Label all possible matches ○ Label matches used for scoring ◉

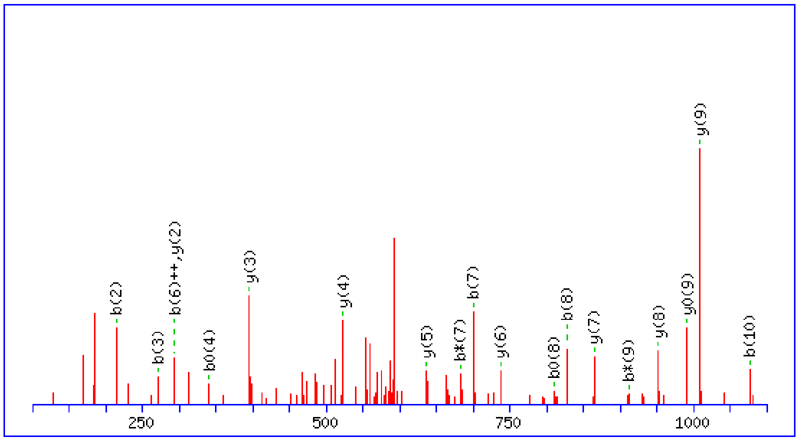

Monoisotopic mass of neutral peptide Mr(calc): 1221.6354
Fixed modifications: Carbamidomethyl (C) (apply to specified residues or termini only)
Ions Score: 57 **Expect:** 0.00042
Matches : 19/110 fragment ions using 30 most intense peaks (help)

#	b	b^{++}	b*	b^{*++}	b^0	b^{0++}	Seq.	y	y^{++}	y*	y^{*++}	y^0	y^{0++}	#
1	115.0502	58.0287	98.0237	49.5155			N							11
2	**214.1186**	107.5629	197.0921	99.0497			V	1108.5997	554.8035	1091.5732	546.2902	1090.5891	545.7982	10
3	**271.1401**	136.0737	254.1135	127.5604			G	***1009.5313***	505.2693	992.5047	496.7560	991.5207	496.2640	9
4	358.1721	179.5897	341.1456	171.0764	340.1615	170.5844	S	***952.5098***	476.7586	935.4833	468.2453	934.4993	467.7533	8
5	486.2307	243.6190	469.2041	235.1057	468.2201	234.6137	Q	***865.4778***	433.2425	848.4512	424.7293	847.4672	424.2373	7
6	587.2784	294.1428	570.2518	285.6295	569.2678	285.1375	T	***737.4192***	369.2132	720.3927	360.7000	719.4087	360.2080	6
7	**700.3624**	350.6849	683.3359	342.1716	682.3519	341.6796	L	***636.3715***	318.6894	619.3450	310.1761	618.3610	309.6841	5
8	**828.4210**	414.7141	811.3945	406.2009	810.4104	405.7089	Q	***523.2875***	262.1474	506.2609	253.6341	505.2769	253.1421	4
9	929.4687	465.2380	912.4421	456.7247	911.4581	456.2327	T	***395.2289***	198.1181	378.2023	189.6048	377.2183	189.1128	3
10	**1076.5371**	538.7722	1059.5106	530.2589	1058.5265	529.7669	F	***294.1812***	147.5942	277.1547	139.0810			2
11							K	147.1128	74.0600	130.0863	65.5468			1

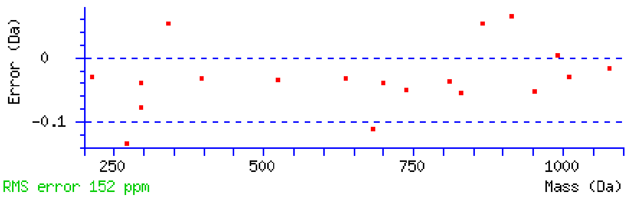

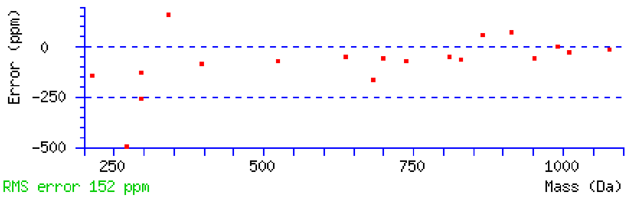

NCBI **BLAST** search of NVGSQTLQTFK
(Parameters: blastp, nr protein database, expect=20000, no filter, PAM30)
Other BLAST web gateways

All matches to this query

Score	Mr(calc):	Delta	Sequence
57.2	1221.6354	-0.0687	NVGSQTLQTFK
11.9	1221.4679	0.0987	GISGKGEGTEK
8.7	1221.5755	-0.0088	RGTLPVDQEK
5.3	1221.6387	-0.0721	NSVLNTATKMK
5.2	1221.5488	0.0178	FSCGNIYSFK
5.2	1221.6758	-0.1091	GSIWLSALTFK
5.2	1221.5795	-0.0129	NFIKSGTTFK
5.2	1221.5795	-0.0129	NFIKSGTTFK
5.2	1221.5795	-0.0129	NFIKTGSTFK
5.2	1221.5795	-0.0129	NFIKTGSTFK

Mascot: http://www.matrixscience.com/

Mascot Search Results

Peptide View

MS/MS Fragmentation of **AAEIASSDSANVSSR**
Found in **PK3CB_HUMAN**, Phosphatidylinositol 4,5-bisphosphate 3-kinase catalytic subunit beta isoform OS=Homo sapiens GN=PIK3CB PE=1 SV=1

Match to Query 447: 1463.579808 from(732.797180,2+) intensity(696801.0000)
Title: Cmpd 72, +MSn(732.7915), 11.8 min (572298)
Local Instrument: ETD-TRAP
Data file 1250.mgf

Click mouse within plot area to zoom in by factor of two about that point
Or, Plot from 400 to 2800 Da Full range
Label all possible matches ○ Label matches used for scoring ◉

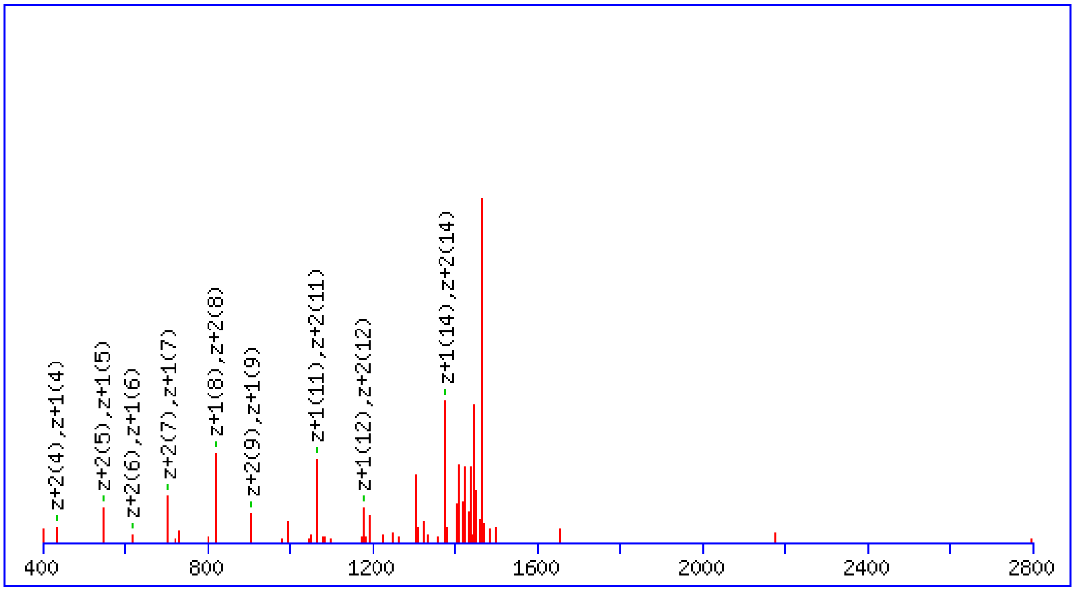

Monoisotopic mass of neutral peptide Mr(calc): 1463.6852
Fixed modifications: Carbamidomethyl (C) (apply to specified residues or termini only)
Ions Score: 90 **Expect:** 2.6e-007
Matches : 18/112 fragment ions using 11 most intense peaks (help)

#	c	c^{++}	Seq.	y	y^{++}	z+1	$z+1^{++}$	z+2	$z+2^{++}$	#
1	89.0709	45.0391	A							15
2	160.1081	80.5577	A	1393.6554	697.3313	***1377.6367***	689.3220	***1378.6445***	689.8259	14
3	289.1506	145.0790	E	1322.6183	661.8128	1306.5995	653.8034	1307.6074	654.3073	13
4	402.2347	201.6210	I	1193.5757	597.2915	***1177.5569***	589.2821	***1178.5648***	589.7860	12
5	473.2718	237.1396	A	1080.4916	540.7494	***1064.4729***	532.7401	***1065.4807***	533.2440	11
6	560.3039	280.6556	S	1009.4545	505.2309	993.4358	497.2215	994.4436	497.7254	10
7	647.3359	324.1716	S	922.4225	461.7149	***906.4037***	453.7055	***907.4116***	454.2094	9

**8**	762.3628	381.6850	**D**	835.3904	418.1989	*819.3717*	410.1895	*820.3795*	410.6934	**8**
**9**	849.3949	425.2011	**S**	720.3635	360.6854	*704.3448*	352.6760	*705.3526*	353.1799	**7**
**10**	920.4320	460.7196	**A**	633.3315	317.1694	*617.3127*	309.1600	*618.3206*	309.6639	**6**
**11**	1034.4749	517.7411	**N**	562.2943	281.6508	*546.2756*	273.6415	*547.2835*	274.1454	**5**
**12**	1133.5433	567.2753	**V**	448.2514	224.6293	*432.2327*	216.6200	*433.2405*	217.1239	**4**
**13**	1220.5753	610.7913	**S**	349.1830	175.0951	333.1643	167.0858	334.1721	167.5897	**3**
**14**	1307.6074	654.3073	**S**	262.1510	131.5791	246.1323	123.5698	247.1401	124.0737	**2**
**15**			**R**	175.1190	88.0631	159.1002	80.0538	160.1081	80.5577	**1**

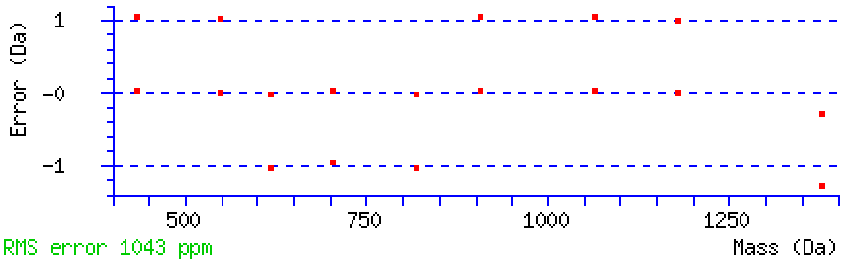

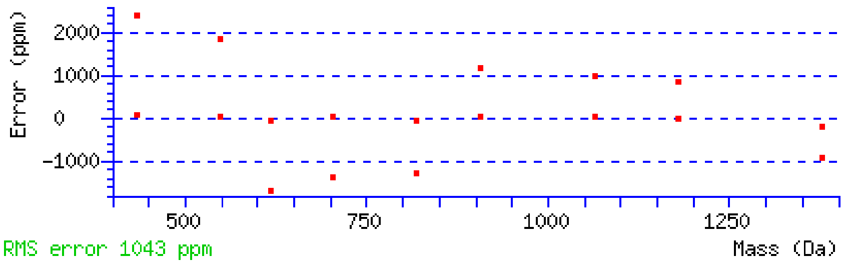

NCBI **BLAST** search of AAEIASSDSANVSSR
(Parameters: blastp, nr protein database, expect=20000, no filter, PAM30)
Other BLAST web gateways

All matches to this query

Score	Mr(calc):	Delta	Sequence
89.7	1463.6852	-0.1054	AAEIASSDSANVSSR
31.0	1462.7425	0.8373	ACLVVSLCISWR
27.4	1462.6470	0.9328	VDGQTGENDMNKR
24.4	1462.6722	0.9076	SLAESIDDALNCR
21.8	1462.4989	1.0809	SQGYPMSAAYGR
18.8	1462.6527	0.9271	ANAINMSDKLYK
18.7	1462.6606	0.9192	AQAGSGYNFLSLR
18.4	1463.6449	-0.0651	SAVEEMEAEEAAAK
18.1	1463.4442	0.1357	AQAEMQSSASSK
17.5	1463.5833	-0.0035	AEAIESSLEVEK

Mascot: http://www.matrixscience.com/

Mascot Search Results

Peptide View

MS/MS Fragmentation of **SVMIDEDMTAR**
Found in **ILK_HUMAN**, Integrin-linked protein kinase OS=Homo sapiens GN=ILK PE=1 SV=2

Match to Query 424: 1298.382298 from(650.198425,2+) intensity(161529.0000)
Title: Cmpd 44, +MSn(650.1984), 12.8 min (561526)
Local Instrument: ESI-TRAP
Data file 1226.mgf

Click mouse within plot area to zoom in by factor of two about that point
Or, Plot from 150 to 1150 Da Full range
Label all possible matches ○ Label matches used for scoring ◉

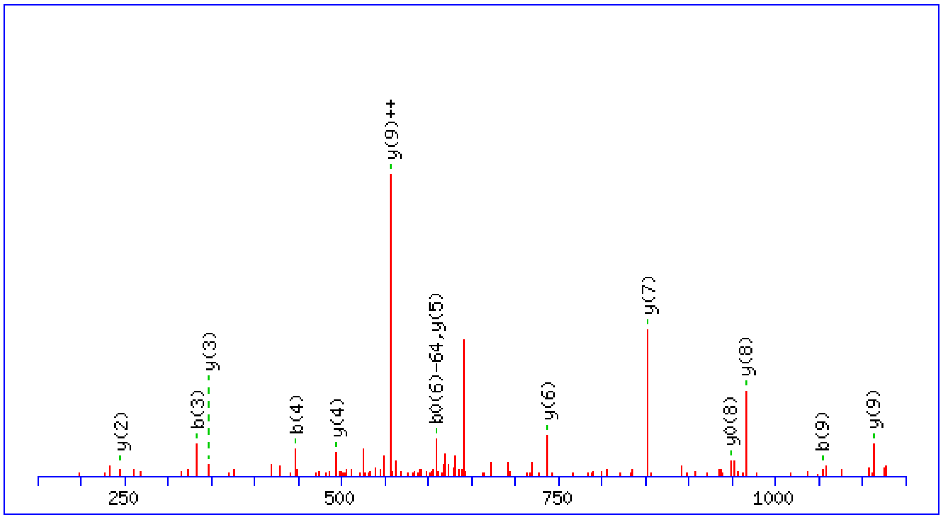

Monoisotopic mass of neutral peptide Mr(calc): 1298.5482
Fixed modifications: Carbamidomethyl (C) (apply to specified residues or termini only)
Variable modifications:
M3 : Oxidation (M), with neutral losses 0.0000(shown in table), 63.9983
M8 : Oxidation (M), with neutral losses 0.0000(shown in table), 63.9983
Ions Score: 63 **Expect:** 9.1e-005
Matches : 14/170 fragment ions using 20 most intense peaks (help)

#	b	b^{++}	b^0	b^{0++}	Seq.	y	y^{++}	y*	y^{*++}	y^0	y^{0++}	#
1	88.0393	44.5233	70.0287	35.5180	S							11
2	187.1077	94.0575	169.0972	85.0522	V	1212.5235	606.7654	1195.4970	598.2521	1194.5129	597.7601	10
3	**334.1431**	167.5752	316.1326	158.5699	M	***1113.4551***	557.2312	1096.4285	548.7179	1095.4445	548.2259	9
4	**447.2272**	224.1172	429.2166	215.1119	I	***966.4197***	483.7135	949.3931	475.2002	948.4091	474.7082	8
5	562.2541	281.6307	544.2436	272.6254	D	***853.3356***	427.1714	836.3091	418.6582	835.3251	418.1662	7
6	691.2967	346.1520	673.2862	337.1467	E	***738.3087***	369.6580	721.2821	361.1447	720.2981	360.6527	6
7	806.3237	403.6655	788.3131	394.6602	D	***609.2661***	305.1367	592.2395	296.6234	591.2555	296.1314	5
8	953.3591	477.1832	935.3485	468.1779	M	***494.2391***	247.6232	477.2126	239.1099	476.2286	238.6179	4
9	**1054.4067**	527.7070	1036.3962	518.7017	T	***347.2037***	174.1055	330.1772	165.5922	329.1932	165.1002	3
10	1125.4439	563.2256	1107.4333	554.2203	A	***246.1561***	123.5817	229.1295	115.0684			2
11					R	175.1190	88.0631	158.0924	79.5498			1

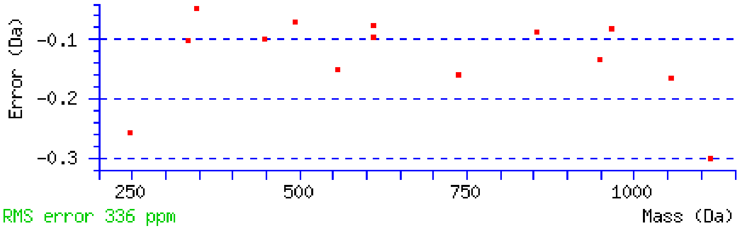

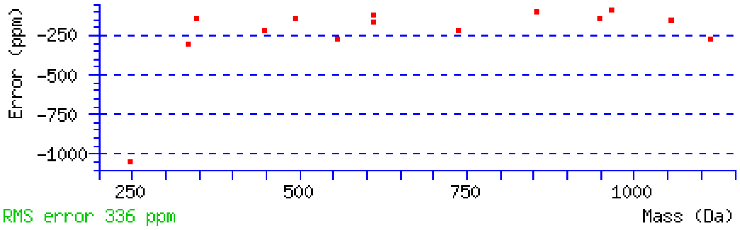

NCBI **BLAST** search of SVMIDEDMTAR
(Parameters: blastp, nr protein database, expect=20000, no filter, PAM30)
Other BLAST web gateways

All matches to this query

Score	Mr(calc):	Delta	Sequence
63.4	1298.5482	-0.1659	SVMIDEDMTAR
12.0	1297.6204	0.7619	AGGFISFNGSWR
9.7	1298.3862	-0.0039	WMESPSCAR
8.1	1297.5551	0.8272	GEASKPTTADNK
8.0	1297.6431	0.7392	EFGLLKPTSAR
7.2	1297.5551	0.8272	GEASKPTTADNK
6.0	1298.5941	-0.2118	DSMILLGSVER
5.3	1297.6279	0.7544	NISNQLSITTK
4.5	1297.6279	0.7544	NISNQLSITTK
4.4	1298.6441	-0.2618	FKAPQYTMAAR

Mascot: http://www.matrixscience.com/

Mascot Search Results

Peptide View

MS/MS Fragmentation of **DVNAAIATIK**
Found in **TBA3E_HUMAN**, Tubulin alpha-3E chain OS=Homo sapiens GN=TUBA3E PE=1 SV=2

Match to Query 261: 1014.466650 from(508.240601,2+) intensity(271611.0000)
Title: Cmpd 300, +MSn(508.2406), 18.6 min (562038)
Local Instrument: ESI-TRAP
Data file 1226.mgf

Click mouse within plot area to zoom in by factor of two about that point
Or, Plot from 100 to 950 Da Full range
Label all possible matches ○ Label matches used for scoring ◉

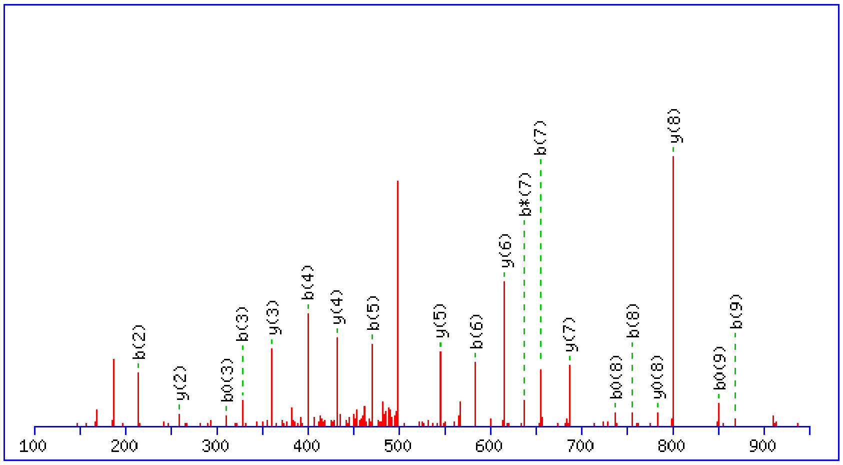

Monoisotopic mass of neutral peptide Mr(calc): 1014.5709
Fixed modifications: Carbamidomethyl (C) (apply to specified residues or termini only)
Ions Score: 82 **Expect:** 1.5e-006
Matches : 20/100 fragment ions using 24 most intense peaks (help)

#	b	b++	b*	b*++	b0	b0++	Seq.	y	y++	y*	y*++	y0	y0++	#
1	116.0342	58.5207			98.0237	49.5155	D							10
2	215.1026	108.0550			197.0921	99.0497	V	900.5513	450.7793	883.5247	442.2660	882.5407	441.7740	9
3	329.1456	165.0764	312.1190	156.5631	311.1350	156.0711	N	801.4829	401.2451	784.4563	392.7318	783.4723	392.2398	8
4	400.1827	200.5950	383.1561	192.0817	382.1721	191.5897	A	687.4400	344.2236	670.4134	335.7103	669.4294	335.2183	7
5	471.2198	236.1135	454.1932	227.6003	453.2092	227.1082	A	616.4028	308.7051	599.3763	300.1918	598.3923	299.6998	6
6	584.3039	292.6556	567.2773	284.1423	566.2933	283.6503	I	545.3657	273.1865	528.3392	264.6732	527.3552	264.1812	5
7	655.3410	328.1741	638.3144	319.6608	637.3304	319.1688	A	432.2817	216.6445	415.2551	208.1312	414.2711	207.6392	4
8	756.3886	378.6980	739.3621	370.1847	738.3781	369.6927	T	361.2445	181.1259	344.2180	172.6126	343.2340	172.1206	3
9	869.4727	435.2400	852.4462	426.7267	851.4621	426.2347	I	260.1969	130.6021	243.1703	122.0888			2
10							K	147.1128	74.0600	130.0863	65.5468			1

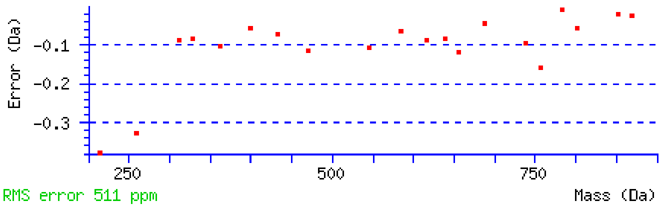

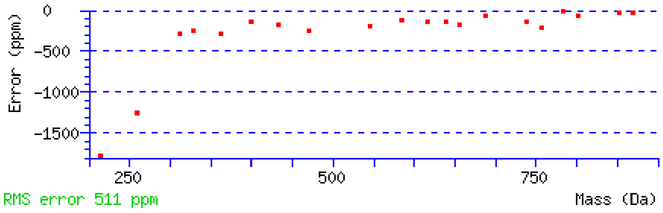

NCBI **BLAST** search of DVNAAIATIK
(Parameters: blastp, nr protein database, expect=20000, no filter, PAM30)
Other BLAST web gateways

All matches to this query

Score	Mr(calc):	Delta	Sequence
81.5	1014.5709	-0.1043	DVNAAIATIK
26.9	1014.5458	-0.0791	GEAAAAALSVR
21.1	1014.5822	-0.1155	AGSLAALEKR
18.5	1014.6073	-0.1407	VDVVNKTLK
17.8	1014.6437	-0.1771	SKLNVLTLK
15.0	1014.5710	-0.1043	QVGEAVATLK
14.7	1014.6437	-0.1771	VKAITAATLK
14.3	1014.5709	-0.1043	KTVSEPNLK
12.7	1014.6437	-0.1771	GTKLVVTGLK
12.0	1013.4616	1.0050	KMSAAEAVK

Mascot: http://www.matrixscience.com/

Mascot Search Results

Peptide View

MS/MS Fragmentation of **VYDVLVLK**
Found in **LY66F_HUMAN**, Lymphocyte antigen 6 complex locus protein G6f OS=Homo sapiens GN=LY6G6F PE=1 SV=2

Match to Query 304: 947.534826 from(474.774689,2+) intensity(1541219.0000)
Title: Cmpd 279, +MSn(474.7747), 23.1 min (578934)
Local Instrument: ESI-TRAP
Data file 1262.mgf

Click mouse within plot area to zoom in by factor of two about that point
Or, Plot from 100 to 950 Da Full range
Label all possible matches Label matches used for scoring

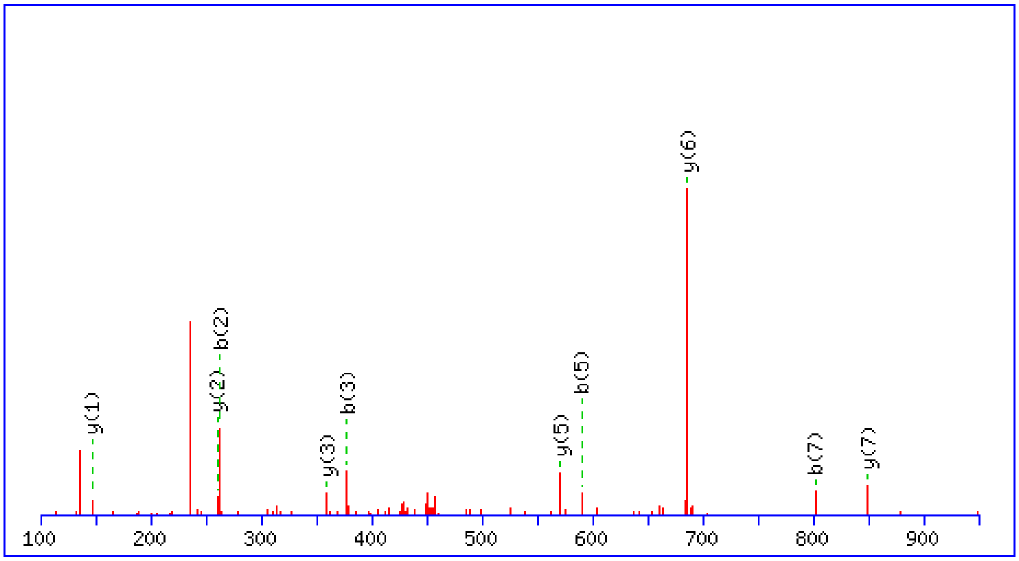

Monoisotopic mass of neutral peptide Mr(calc): 947.5692
Fixed modifications: Carbamidomethyl (C) (apply to specified residues or termini only)
Ions Score: 40 **Expect:** 0.02
Matches : 10/56 fragment ions using 22 most intense peaks (help)

#	b	b^{++}	b^{0}	b^{0++}	Seq.	y	y^{++}	y*	y^{*++}	y^{0}	y^{0++}	#
1	100.0757	50.5415			V							8
2	**263.1390**	132.0731			Y	***849.5080***	425.2577	832.4815	416.7444	831.4975	416.2524	7
3	**378.1660**	189.5866	360.1554	180.5813	D	***686.4447***	343.7260	669.4182	335.2127	668.4341	334.7207	6
4	477.2344	239.1208	459.2238	230.1155	V	***571.4178***	286.2125	554.3912	277.6992			5
5	**590.3184**	295.6629	572.3079	286.6576	L	472.3493	236.6783	455.3228	228.1650			4
6	689.3869	345.1971	671.3763	336.1918	V	***359.2653***	180.1363	342.2387	171.6230			3
7	**802.4709**	401.7391	784.4604	392.7338	L	***260.1969***	130.6021	243.1703	122.0888			2
8					K	***147.1128***	74.0600	130.0863	65.5468			1

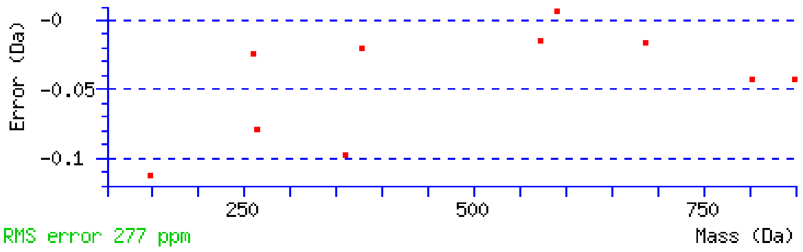

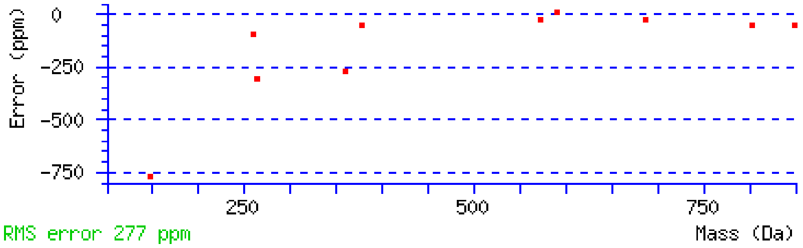

NCBI **BLAST** search of VYDVLVLK
(Parameters: blastp, nr protein database, expect=20000, no filter, PAM30)
Other BLAST web gateways

All matches to this query

Score	Mr(calc):	Delta	Sequence
40.0	947.5692	-0.0343	VYDVLVLK
15.5	947.4600	0.0748	VYSTTSYK
11.7	947.4242	0.1106	VRSSLVK
10.2	946.4525	1.0824	SFSAKSLK
8.8	947.3493	0.1855	MMSGSDIK
7.5	946.5196	1.0152	RTTLVDSR
7.5	946.4356	1.0992	SSSDPQAQK
6.8	947.3857	0.1491	VMMKESK
6.8	947.5076	0.0272	SFLNKDPK
6.5	947.3493	0.1855	MMSGSDIK
